# Advances in delivery methods of *Arthrospira platensis* (spirulina) for enhanced therapeutic outcomes

**DOI:** 10.1080/21655979.2022.2100863

**Published:** 2022-08-10

**Authors:** Omar Ashraf ElFar, Nashiru Billa, Hooi Ren Lim, Kit Wayne Chew, Wai Yan Cheah, Heli Siti Halimatul Munawaroh, Deepanraj Balakrishnan, Pau Loke Show

**Affiliations:** a School of Pharmacy, Faculty of Science and Engineering, University of Nottingham Malaysia, Jalan Broga, Semenyih, Malaysia; b Department of Pharmaceutical Sciences, College of Pharmacy, QU Health, Qatar University, Doha, Qatar; c Department of Chemical and Environmental Engineering, Faculty of Science and Engineering, University of Nottingham Malaysia, Jalan Broga, Semenyih, Malaysia; d School of Energy and Chemical Engineering, Xiamen University Malaysia, Sepang, Malaysia; e College of Chemistry and Chemical Engineering, Xiamen University, Xiamen, China; f Centre of Research in Development, Social and Environment (SEEDS), Faculty of Social Sciences and Humanities,Universiti Kebangsaan Malaysia, 43600 UKM Bangi, Selangor Darul Ehsan, Malaysia; g Study Program of Chemistry, Department of Chemistry Education, Universitas Pendidikan Indonesia, Bandung, Indonesia; h Department of Mechanical Engineering, Jyothi Engineering College, Thrissur, India

**Keywords:** *Arthrospira platensis*, *Arthrospira maxima*, formulation development, drug delivery, delay release, polymer optimization, controlled release, liposomes, femtoscale

## Abstract

*Arthrospira platensis* (*A. platensis*) aqueous extract has massive amounts of natural products that can be used as future drugs, such as C-phycocyanin, allophycocyanin, etc. This extract was chosen because of its high adaptability, which reflects its resolute genetic composition. The proactive roles of cyanobacteria, particularly in the medical field, have been discussed in this review, including the history, previous food and drug administration (FDA) reports, health benefits and the various dose-dependent therapeutic functions that *A. platensis* possesses, including its role in fighting against lethal diseases such as cancer, SARS-CoV-2/COVID-19, etc. However, the remedy will not present its maximal effect without the proper delivery to the targeted place for deposition. The goal of this research is to maximize the bioavailability and delivery efficiency of *A. platensis* constituents through selected sites for effective therapeutic outcomes. The solutions reviewed are mainly on parenteral and tablet formulations. Moreover, suggested enteric polymers were discussed with minor composition variations applied for better storage in high humid countries alongside minor variations in the polymer design were suggested to enhance the premature release hindrance of basic drugs in low pH environments. In addition, it will open doors for research in delivering active pharmaceutical ingredients (APIs) in femtoscale with the use of various existing and new formulations.

**Abbrevations:** SDGs; *Sustainable Development Goals*, IL-4; *Interleukin-4*, HDL; *High-Density Lipoprotein*, LDL; *Low-Density Lipoprotein*, VLDL; *Very Low-Density Lipoprotein*, C-PC; *C-Phycocyanin*, APC; *Allophycocyanin*, PE; *Phycoerythrin*, COX-2; *Cyclooxygenase-2*, RCTs; *Randomized Control Trials*, TNF-α; *Tumour Necrosis Factor-alpha*, γ-LFA; *Gamma-Linolenic Fatty Acid*, PGs; *Polyglycans*, PUFAs: *Polyunsaturated Fatty Acids*, NK-cell; *Natural Killer Cell*, FDA; *Food and Drug Administration*, GRAS; *Generally Recognized as Safe*, SD; *Standard Deviation*, API; *Active Pharmaceutical Ingredient*, DW; *Dry Weight*, IM; *Intramuscular*, IV; *Intravenous*, ID; *Intradermal*, SC; *Subcutaneous*, AERs; *Adverse Event Reports*, DSI-EC; *Dietary Supplement Information Executive Committee*, cGMP; *Current Good Manufacturing Process, A. platensis; Arthrospira platensis, A. maxima; Arthrospira maxima*, Spirulina sp.; *Spirulina* species, *Arthrospira; Spirulina*, Tecuitlatl; *Spirulina*, CRC; *Colorectal Cancer*, HDI; *Human Development Index*, Tf; *Transferrin*, TfR; *Transferrin Receptor*, FR; *Flow Rate*, CPP; *Cell Penetrating Peptide*, SUV; *Small Unilamenar Vesicle*, LUV; *Large Unilamenar Vesicle*, GUV; *Giant Unilamenar Vesicle*, MLV; *Multilamenar Vesicle*, COVID-19; *Coronavirus-19*, PEGylated; *Stealth*, PEG; *Polyethylene Glycol*, OSCEs; *Objective Structured Clinical Examinations*, GI; *Gastrointestinal Tract*, CAP; *Cellulose Acetate Phthalate*, HPMCP, *Hydroxypropyl Methyl-Cellulose Phthalate*, SR; *Sustained Release*, DR; *Delay Release*, Poly(MA-EA); *Polymethyl Acrylic Co-Ethyl Acrylate, f*-DR L-30 D-55; *Femto-Delay Release Methyl Acrylic Acid Co-Ethyl Acrylate Polymer*, MW; *Molecular Weight*, T_g_; *Glass Transition Temperature*, SN_2_; *Nucleophilic Substitution 2*, EPR; *Enhance Permeability and Retention*, VEGF; *Vascular Endothelial Growth Factor*, RGD; *Arginine-Glycine-Aspartic Acid*, VCAM-1; *Vascular Cell Adhesion Molecule-1*, P; *Coefficient of Permeability*, PES; *Polyether Sulfone*, pH_e_; *Extracellular pH*, ζ-potential; *Zeta potential*, NTA; *Nanoparticle Tracking Analysis*, PB; *Phosphate Buffer*, DLS; *Dynamic Light Scattering*, AFM; *Atomic Force Microscope*, Log P; *Partition Coefficient*, MR; *Molar Refractivity*, tPSA; *Topological Polar Surface Area*, C log P; *Calculated Partition Coefficient*, CMR; *Calculated Molar Refractivity*, Log S; *Solubility Coefficient*, pka; *Acid Dissociation Constant*, DDAB; *Dimethyl Dioctadecyl Ammonium Bromide*, DOPE; *Dioleoylphosphatidylethanolamine*, GDP; *Good Distribution Practice*, RES; *Reticuloendothelial System*, PKU; *Phenylketonuria*, MS; *Multiple Sclerosis*, SLE; *Systemic Lupus Erythematous*, NASA; *National Aeronautics and Space Administration*, DOX; *Doxorubicin*, ADRs; *Adverse Drug Reactions*, SVM; *Support Vector Machine*, MDA; *Malondialdehyde*, TBARS; *Thiobarbituric Acid Reactive Substances*, CRP; *C-Reactive Protein*, CK; *Creatine Kinase*, LDH; *Lactated Dehydrogenase*, T2D; *Type 2 Diabetes*, PCB; *Phycocyanobilin*, PBP; *Phycobiliproteins*, PEB; *Phycoerythrobilin*, DPP-4; *Dipeptidyl Peptidase-4*, MTT; *3-(4,5-dimethylthiazol-2-yl)-2,5-diphenyl-2H-tetrazolium bromide*, IL-2; *Interleukin-2*, IL-6; *Interleukin-6*, PRISMA; *Preferred Reporting Items for Systematic Reviews and Meta-Analyses*, STATA; *Statistics*, HepG2; *Hepatoblastoma*, HCT116; *Colon Cancer Carcinoma*, Kasumi-1; *Acute Leukaemia*, K562; *Chronic Leukaemia*, Se-PC; *Selenium-Phycocyanin*, MCF-7; *Breast Cancer Adenocarcinoma*, A375; *Human Melanoma*, RAS; *Renin-Angiotensin System*, IQP; *Ile-Gln-Pro*, VEP; *Val-Glu-Pro*, M_pro_; *Main Protease*, PL_pro_; *Papin-Like Protease*, BMI; *Body Mass Index*, IC_50_; *Inhibitory Concentration by 50%*, LD_50_; *Lethal Dose by 50%*, PC12 Adh; *Rat Pheochromocytoma Cells*, RNS; *Reactive Nitrogen Species*, Hb1Ac; *hemoglobin A1c*.

## Highlights

- Increase awareness of the impact and multi-disciplinary up-to-date roles of *A. platensis* on human lives and the importance of having further research on microalgae.
- Soliciting a critical analysis study on *A. platensis* biocomposition for drug delivery research.
- Insights on the correlation between ionization and drug bioavailability in specific sites in the human body.
- Offering solutions for improvising an optimized ‘Advanced Spirulina Dosage Forms’ products to maximize *A. platensis* therapeutic/pharmacological outcomes.
- Insights on existing biomaterials for optimization.

## Graphical Abstract

**Figure:**
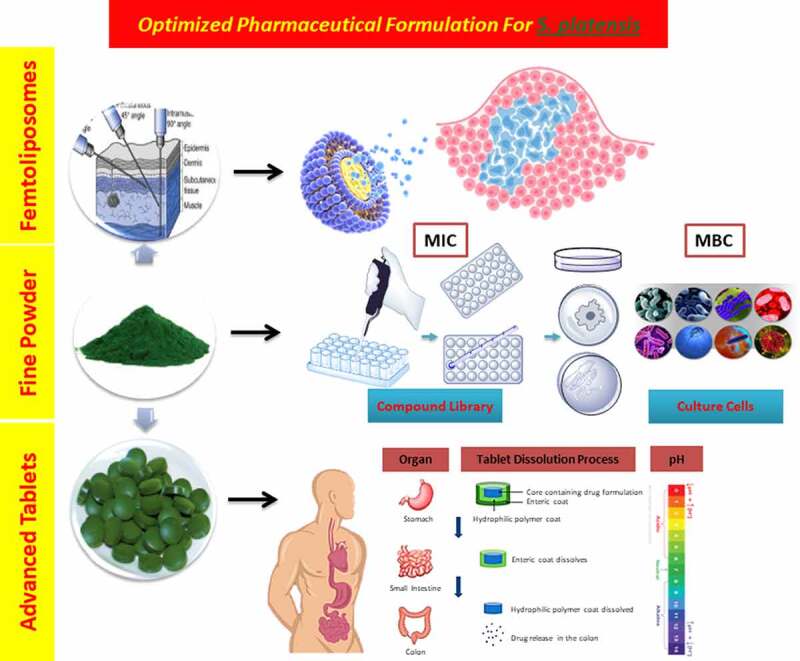

## 1. Introduction

The practise of drug delivery has changed dramatically in the past few decades [1], and even greater changes are anticipated. Many drugs, even those discovered using the most advanced molecular biology strategies, have unacceptable side effects due to the drug interaction [2] with healthy tissues, which are not the target of the expected drug. Without effective delivery, this would affect the ability of the drug to exert its functions; for instance, poor absorption means that the required dose to exert its function is incomplete.

Inefficient drug delivery leads to the rise of ‘off-targets’ [3], which is known to be one of the major causes of adverse drug reactions (ADRs) commonly [4] considered as side effects where their activity is not required in particular places in the body system, which is obvious due to the fact that drugs entering the systemic circulation are distributed freely. The use of similarity ensemble approach and relating protein-based targets using *insilico* software by setting up the chemical structures similarities among their ligands [3] have identified the side effects of any drug through the virtual screening of compounds by running cheminformatics and bioinformatics analysis tools with applied Altman’s algorithm and support vector machine (SVM) functions [5], have provided scientists with less decision time and cost to predict any potential side effects of drugs either alone or in combination with other drugs. Also, to verify how promising the drug design was before the actual practical work.

The most challenging and important parameters required to be considered in drug delivery systems that should be considered such as pressure, enzymatic activity [6], pH [7], solubility, molecular size and reticuloendothelial system [8]. The aim of drug targeting has always been to direct the drug to the target tissue either at a cellular level or subcellular level where its activity is required [1,4,9,10]. This paper presents two new coating formulations in existing drug delivery systems: the first one is for *A. platensis* tablets, where a double layer of polymers [11] will be added on top of the core. The second formulation is a parenteral formulation, especially for intramuscular use, whereby the extract will be delivered in PEGylated femtoliposome carriers to the specified target.

One of the potential natural products that could be tested with these optimized formulations is *A. platensis*, which is categorized under the micro-algae family [12]. This product was chosen because it has scientifically proven its promising roles in the medical field in fighting against lethal diseases [13]. Algae are photosynthetic living organisms that convert light energy from the sun into chemical energy [14]. These organisms have attracted considerable attention and interest in developing bioactive compounds, food, and their use in environmental cleaning [15]. However, the revolution in the algae industry has shown that there is a significant gap in the realization of the increasing energy demands and the achievement of environmental sustainable development goals (SDGs) via the use of algae [16,17].

These organisms are classified taxonomically in Bacteria’s kingdom; phylum of Cyanobacteria; order of Oscillatoriales; family of Phormidiaceae and genus of Arthrospira [18]. The cyanobacterium species *Athrospira maxima* (*A. maxima*) and *platensis* (*A. platensis*) are currently the most commonly harvested species for their maximum protein content known as ‘Spirulina’ compared to other species. The production of Spirulina have many benefits due to its dense complexity content from variant natural products, for instance, phycocyanin, lipids, carotenoids, and fatty acids for implementation in cosmetics, organic food, food supplements, pharmaceuticals, and fuel production that has shown a great benefit to the human’s health [14,19,20].

The natural products extracted from plants and algae have been reported in portraying essential roles. This was achieved by the renowned process scheme of phytochemical extraction of natural products in drug discovery via the process of isolation, characterization by a combination of screening, imaging mass spectrometry and computational chemistry (cheminformatics) and utilization of derived bioactive compounds from leads, which is regarded as drug candidates [21]. Successful identification of pharmacodynamic activities in chosen bioactive compounds is further processed for pre-clinical and pharmacokinetics profiling.

Examples of well-known natural products extracted from microalgae microbiomes and used in therapeutics is called phycocyanin, a blue pigment and water-soluble biliprotein extracted from the *A. platensis* [22]. It has great medical and medicinal properties, a powerful anti-oxidant and anti-inflammatory remedy. Moreover, it has been used as a nutritional supplement because it is enriched content with proteins, carbohydrates and lipids [23]. Besides microalgae, plants are known for their vast source of novel bioactive natural products such as the antitumour agents’ maytansine, paclitaxel, camptothecin, podophyllotoxin [24], and turmeric spice that comes from the turmeric plant. Curcumin is an active ingredient extracted from turmeric that possesses potent anti-oxidant, anti-inflammatory and anti-cancerous properties [25]. The moral behind providing such examples of successful medicinal natural products is to show how far natural products could reach, which is what this paper doing to throw more attention to microalgae’s capability on curing many diseases and this was proven in several *in silico, in vitro*, and *in vivo* studies. The way microalgae survive is just the same way plants do through photosynthesis, which means that microalgae could become a very crucial source of medicinal products as well.

The main aim of this paper is to show appreciation to algae researchers and activists to increase awareness through digital marketing about the multi-therapeutic effects that Spirulina exerts. To throw lights on the pool of metabolites that could be developed for future biomedical and pharmaceutical applications.

There are two objectives to investigate this aim: the first objective is to deliver the *A. platensis* orally via a double-coated layer tablet formulation. The first coating layer is a femtopolymer layer, which is responsible to delay the release of the drug from the stomach, mainly focusing on improving the premature release hindrance of basic drugs in acidic environments. The second coating layer is responsible for improving the pulsatile release of the extract at zero kinetic order in the ileocolonic segments, making both release and bioavailability more reliable and maximized. The second objective is to offer a new formulation to deliver *A. platensis* extract in femtoliposomes carriers and each carrier loaded in femtolitres for intramuscular (IM) parenteral formulation. This procedure hypothetically would improve the bioavailability, flowability and the instant effect of the carriers within the target of interest.

## 2. Morphology

**Figure 1.**
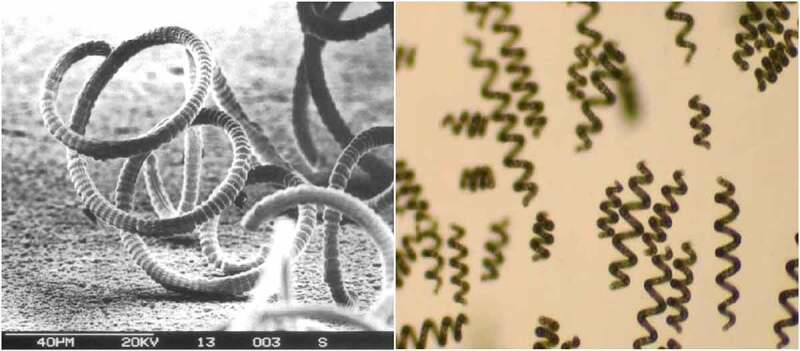
Captured microscopic images of *A. platensis.*

Spirulina (*Arthrospira*) genus name is derived from the helical or spiral nature of its filaments, which is a cyanobacterium categorized under the Oscillatoriaceae family [26]. The light microscope single plane specimen shows the transverse cross-walls and the blue-green non-heterocystous filament, as illustrated in Figure 1 (left hand-side) [27]. Composed of vegetative cells that undergo binary fission. Filaments are solitary, free-floating and display gliding motility. Also, *A. platensis* is a gram-negative cyanobacterium because of its thin peptidoglycan width with a lack of membrane-bound organelles. This is observed using SEM to capture the width and more comprehensive information on the sheath [28,29], as illustrated in Figure 1 (right hand-side) [27].

Environmental factors such as chemical conditions and temperature may affect the helix geometry and cause drastic alterations. After the strain has been physically converted to the straight form due to the induction of chemical treatments such as radiation from UV and other chemicals, or even naturally; the helical pattern [26] does not return because of the mutation affecting the trichomes during development [26,28]. The mutated form of the spiral reflects on the poor environment the species were placed in for growth. The cellular, thin-walled nature of the *A. platensis* outer layer gives it an advantage of easier digestibility of approximately 85–95% [29] than other algae [30]. The cell wall is a soft membrane composed mainly of a peptidoglycan and lipopolysaccharide composition, consisting of various layers. *Athrospira* cells have several inclusions, including phycobilisomes integrated into thylakoid membranes, carboxysomes, ribosomes, DNA fibrils, gas vacuoles, polyglycans, polyphosphates, and cyanophycin granules [31].

The species morphology is maintained and changed in the medium of an alkaline environment, saline water with more than 30 g/L, and a high pH range from 8.5 to 11.0. *A. platensis* encourages this medium, particularly at altitude, where solar radiation is intense in the tropics, expanding and maintaining its rapid growth efficiency. *A. platensis* is an obligatory photoautotrophic species; it cannot develop in darkness on organic carbon compound mediums without sunlight [29].

## 3. Biochemical composition

A critical study and analysis of the *A. platensis* composition are one of the most important keys to successful drug delivery since it is a composition that is delivered at once as a whole. Identifying each compound in the extract composition would give insights into the possible sites for dissolution of the extract or isolated potential drug candidates for maximal therapeutic outcomes because isolated individual substances’ physico-chemical parameters are completely different from the same substance in a composition. For instance, the best candidates for colon targeting are active ingredient agents that display less absorption from the upper portion of the gastrointestinal tract than the intestine. In this section, a full screening of *A. platensis* extract composition has been performed and adopted from analytical scientific journals to determine the exact quantities of each substance and the cumulative characteristics of the extract.

*A. platensis* is one of the most hopeful microalgae species, protein-enriched with essential amino acids, polyunsaturated fatty acids (PUFAs) and vitamins, in particular, B12 and provitamin A (β-carotenes), and minerals, in particular iron [19,29,32,33]. This also includes a high phenol, tocopherols and linolenic acid contents [34], minerals and several phytonutrients. In addition, *A. platensis* has no cellulose surfaces, making it edible and easy to digest [34]. *A. platensis* has a large amount of protein, with a dry weight of 60–70% [29,35–38]. This is a complete protein surplus that includes all important amino acids that the human body requires, including leucine, isoleucine, and valine, with small amounts of methionine, cystine, and lysine [32,33] in comparison with normal proteins such as beef, egg or milk.

Two phycobiliproteins are produced by *A. platensis*. A major and minor pigment incorporated in *A. platensis* total composition represented significant findings: C-phycocyanin (C-PC) and Allophycocyanin (APC). Quantitatively, both pigments contribute to an approximately 10:1 ratio, respectively. C-PC is the most abundant phycobiliprotein present and varies in its level depending on the growth and development conditions that collectively sum up almost 20% of the cell’s dry weight [26] and according to Sarada *et al.* [39,40] accounted for 19.4 ± 0.4 mg of C-PC per 100 mg of Spirulina powder. The chromophore present, phycocyanobilin (PCB), accommodates the two homologues proteins, C-PC and APC, bind to the PCB chromophore active site for physiological activities. The subject of debate in *A. platensis* is about the potential of the newly discovered and presence of a third phycobiliprotein, which is the red phycoerythrin. According to scientifically performed studies discovered that small amounts of phycoerythrin were found in *A. platensis*. However, some other studies stated that phycoerythrin was not detected, which is still debatable [41].

*A. platensis* has a high amount of polyunsaturated fatty acids (PUFAs), 1.5–2.0% out of 5–6% of total lipid [33,42,43]. In particular, it is rich in γ-linolenic acid (36% of total PUFAs), and also provides γ-linolenic acid (ALA), linoleic acid (LA, 36% of total), stearidonic acid, eicosapentaenoic acid, docosahexaenoic acid, and arachidonic acid [29,33,44–46]. In fact, it also produces fairly high vitamin amounts of B1 (thiamine), B2 (riboflavin), B3 (nicotinamide), B6 (pyridoxine), B9 (folic acid), B12 (cyanocobalamin), vitamin C (ascorbic acid), vitamin D (cholecalciferol), which scientifically proves its resolute anti-oxidant property [26,28,29,33,47]. About 7% of all the essential minerals are available in *A. platensis*, including potassium, calcium, chromium, copper, iron, magnesium, manganese, phosphorus, selenium, sodium, and zinc [26,28,29,32,33,44,45,48].

Furthermore, *A. platensis* contains many pigments, including chlorophyll-*a*, xanthophyll, beta-carotene, echinenone, myxoxanthophyll, zeaxanthin, canthaxanthin, diatoxanthin, 3-hydroxyechinenone, beta-cryptoxanthin, oscillaxanthin, C-phycocyanin, and allophycocyanin [29,35]. Besides, *A. platensis* contains about 13.5% carbohydrates [19,29,32,33], which is mainly composed of glucose, along with rhamnose, mannose, xylose, galactose, and two unusual sugars, including 2-O-methyl-l-rhamnose and 3-O-methyl-l-rhamnose [29]. The biochemical composition of *A. platensis* is critically summarized in Table 1, adapted from scientific journals referenced in the paragraphs mentioned above.

**Table 1.** Critical biochemical composition profile analysis of A. platensis.

*Arthrospira platensis* Biochemical Composition Profile		
Content	Amount/Value	Unit
Carbohydrates	15.0-25.0	%
Proteins	65.0-71.0	%
Amino Acids		
Alanine	7.7-46.6	mg/g
Arginine	7.9-47.6	mg/g
Aspartic Acid	12.1-72.8	mg/g
Cysteine	0.9-5.6	mg/g
Glutamic Acid	4.1-84.4	mg/g
Glycine	5.3-31.9	mg/g
Histidine	2.5-15.0	mg/g
Isoleucine	5.4-32.6	mg/g
Leucine	8.2-48.9	mg/g
Lysine	4.4-26.2	mg/g
Methionine	2.2-13.3	mg/g
Phenylalanine	4.5-26.1	mg/g
Praline	4.1-24.7	mg/g
Serine	4.4-26.5	mg/g
Threonine	4.7-28.1	mg/g
Tryptophan	1.4-8.5	mg/g
Tyrosine	4.0-23.8	mg/g
Valine	6.2-37.4	mg/g
Fibers	4.0-7.0	%
Lipids	6.0-12.0	%
Fatty Acids		
Omega 6		
Gamma Linolenic	30.00	mg/g
Essential Linolenic	33.00	mg/g
Dihomogamma Linolenic	1.59	mg/g
Omega 3		
Alpha Linolenic	0.04	mg/g
Docosahexaenoic	0.04	mg/g
Palmitoleic	5.90	mg/g
Oleic	0.50	mg/g
Erucic	0.07	mg/g
Moisture	4.0-5.0	%
Minerals		
Calcium	1.68	mg/g
Magnesium	2.55	mg/g
Iron	0.52	mg/g
Phosphorous	9.18	mg/g
Potassium	18.30	mg/g
Sodium	10.98	mg/g
Boron	0.30	mg/g
Manganese	0.19	μg/g
Zinc	0.20	μg/g
Copper	0.30	μg/g
Molybdenum	0.30	μg/g
Phytopigments		
Total Carotenoids	0.551	%
Beta-carotenoids	0.243	%
Xanthophylls	0.271	%
Zeaxanthin	0.128	%
Chlorophyll	1.472	%
Phycocyanin	14.18	%
Water-soluble vitamins		
B-Complex vitamins		
Vitamin B1 (Thiamine)	238.00	mg/g
Vitamin B2 (Riboflavin)	99.00	mg/g
Vitamin B3 (Niacin)	3.67	mg/g
Vitamin B5 (Pantothenic Acid)	3.40	mg/g
Vitamin B6 (Pyridoxine)	13.20	mg/g
Vitamin B9 (Folate)	94.00	μg/g
Vitamin B12 (Cyanocobalamin)	6.60	μg/g
Vitamin H (Biotin)	1.00	mg/g
Choline	66.00	mg/g
Vitamin C (Ascorbic Acid)	58.80	mg/g
Fat-soluble vitamins		
Vitamin A (as Beta Carotene)	29.00	μg/g
Vitamin E (D-atocopherol)	5.00	mg/g
Vitamin K (Phytomenadione)	25.20	μg/g
Alpha Carotene	7.50	μg/g
Beta Carotene	1900.00	μg/g
Lutein and Zeaxanthin	126.00	μg/g
Energy	2.9	Kcal/g

### 3.1. Content profile

## 4. Pharmacovigilance review

The photosynthetic planktonic cyanobacterium shapes like broad colonies of tropical and subtropical water bodies called *A. platensis* consisting of significant concentrations of salts, such as carbonate and bicarbonate with an alkaline pH of 9.5 [14]. *A. platensis* is now classified as General Recognized As Safe (GRAS) by the US Food and Drug Administration (FDA) since 2003, which belongs to the substances that are accepted to be regarded as a dietary health supplement [34,49]. Usually, adults recommended dosage to consume *A. platensis*; is in the range of 3–10 g per day, while the maximum daily intake should not exceed 30 g [41,50].

Many toxicological studies have scientifically demonstrated the safety of *A. platensis* [34], whereby it proved that there is no cyanobacterial toxin reports in *A. platensis* species have been reported to date. *A. platensis* does not typically produce toxins. However, there is a high probability that other cyanobacteria could contaminate the outdoor arena during cultivation. Nevertheless, the proper monitoring and management of *A. platensis* monoculture are unlikely to be a problem [29]. There was a variety of checked pieces of evidence in adverse event reports (AERs). However, there is limited information available regarding *A. platensis*. The dietary supplement information executive committee (DSI-EC) unanimously voted for a ‘Class A’ safety assignment for *A. maxima* and *A. platensis*, indicating that the available evidence does not indicate any serious risk to health or public health [51].

This alga is a significant staple diet for humans and has been used without serious side effects as a dietary supplement enriched with protein and vitamins [52]. Also, it provides a wide variety of specific nutrient components, such as peptides, sugars, lipids, pigments, and other essential helpful trace elements, compared to other conventional plants for the human diet [16,53]. *A. maxima* and *A. platensis* are the two most common types of Spirulina species. The quality of micro and macronutrients is substantial. The dry weight chemical composition is made of protein, carbohydrate, and vitamins such as pro-vitamin A, vitamin C, and vitamin E. Besides, minerals including iron, calcium, chromium, copper, magnesium, manganese, phosphorus, potassium, sodium, and zinc. In addition, essential fatty acids like g-linolenic acid (GLA) and pigments such as chlorophyll-a, phycocyanin, and carotenes are also present. Spirulina is also used in the care of cosmetics, medications, and contaminated water treatment. On top of this, the cell wall is composed of polysaccharides and can be readily absorbed by the human body, with 86% of the content digestion [14,26,29,34].

Besides, researchers have identified *A. platensis* pharmacological properties, which is the reason behind identifying *A. platensis* safest dose and its effect in the long term. Identifying those properties clinically has increased *A. platensis* safety, such as anti-oxidant, pain-relief, anti-inflammatory, and brain-protective properties [34]. For example, Hutadilok-Towatana *et al.* [54] conducted toxicological studies on mice using *A. platensis* aqueous extract. The researcher collected the mice’s blood samples after reconstituting a single dose of up to 6 g kg^−1^ body weight and administered orally 5 mL kg^−1^. After 7 days from the administration day, the author claimed that *A. platensis* did not cause any toxicity and recorded it as the safest dose of *A. platensis* with no issues in the long term since the biopsy and autopsy reports were fine. The same study (up-to-date) was conducted to assure its safety by Ngu *et al.* [55], but was *in vitro* and reported that after the administration of more than 5 mg.mL^−1^ of *A. platensis* aqueous extracts on cultured rat pheochromocytoma (PC12 Adh) cells [56], the cell viability was more than 50%. In addition, Sagara *et al.* [57] tested the same extract on brain cells and found that the brain cells were protected against iron toxicity due to the excessive accumulation, whereby the author concluded that it acts as a defensive layer against particular neurodegenerative disorders due to its saturated content of polyphenolic compounds.

Furthermore, *A. platensis* powder contains protein, which is considered a complete source of high-quality protein and is often compared to eggs for the amount of protein per gram [32,33,48]. Besides, it contains many vitamins, for instance, vitamin B1, also known as Thiamine. This vitamin is necessary for the digestion of fats and proteins. *A. platensis* provides a great energy surplus for eye health, brain function and for enhancing nerve functions. *A. platensis* contains iron, which is the most recommended food for vegetarians and vegans to avoid anemia and increase vitality [58]. Even omnivores have a highly absorbable form of iron; almost 1.5-2 mg of iron are absorbed out of 10 g of *A. platensis* powder dose; moreover, *A. platensis* powder is gentle on the digestive system [59]. *A. platensis* powder also contains a significant amount of calcium, relatively over 26 times the calcium in milk [60].

## 5. Pharmacotherapy review

*A. platensis* aqueous extract has been reported to possess multiple therapeutic effects, including immunomodulation and booster, cholesterol depletion, anti-viral, anti-oxidant, anti-cancer, anti-allergic, anti-inflammatory, reducing blood glucose levels and anti-microbial activities [38,52] against particular infections. However, all the activities found in primary literature reviews were all dose-dependent, which means that each isolated and purified compound from *A. platensis* would have an effect if the ‘proof-of-concept’ of the natural product suggested by Cos *et al.* [61] is applied to each mentioned potential compound is pharmacologically effective to prove that it is significant, valid and cost-effective (from a macroscale production perspective). Since part of lead analogues selection and optimization are mainly based on the selected disease target and delivery pathway to the target to ensure a maximal effect (the lower the ED_50_ and higher LD_50_ (the higher the therapeutic index, $TI=\frac{LD_{50}}{ED_{50}}$, the safer the drug) reflects the drug safety and effectiveness). All *in vitro* data should be close enough to *in vivo* results in order to verify them as promising medicinal drugs to be considered by the FDA for evaluation (backed-up with clinical reports).

**Figure 2.**
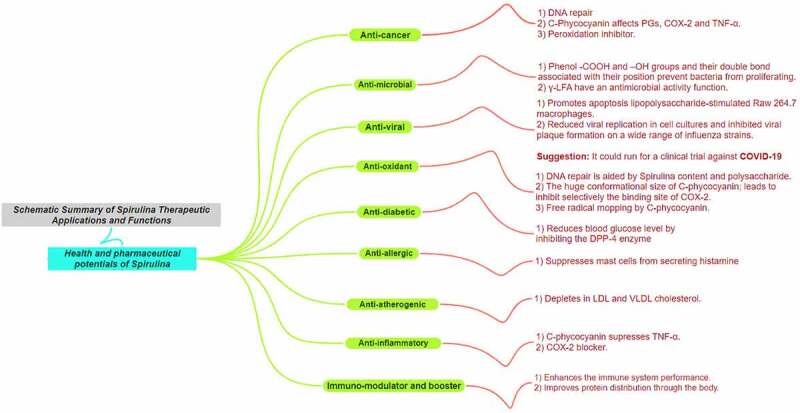
A. platensis broad functions mind map.

*A. platensis* is expected to be one of the most used pools of natural products to generate desireful drugs for future major applications due to its high adaptability and low consumption of resources for survival and that would let us anticipate how strong alga genes and rapidly responsive is it toward the environment, which would definitely reflect on the strong chemical compounds it generates for survival against invaders or toxins. It was further investigated by Plaza *et al.* [62]. by demonstrating insights into ‘Innovative natural functional ingredients from microalgae’, including reliable extraction methods. A summary mind map about the broad functions that *A. platensis’* exerts [63] is illustrated in Figure 2. All of the mentioned functions are dose-dependent (dose–response % graph is recommended) and that was achieved after successful extraction and purification for further assessment. One of the most reliable methodologies for the extraction, isolation, and purification of natural products derived from *A. platensis* are in the following references: Organic and inorganic compounds [64], Chlorophyll-a [65], C-phycocyanin (C-PC) [40,66,67], α-Glucans [68] and lastly a creative approach in using eutectic solvents for the extraction of natural products [69] for *in vivo* and *in vitro* studies.

In the upcoming paragraphs, the various effects possessed by fresh biomass of *A. platensis* extracts against particular biomarkers and its benefits for appreciation will be discussed for its application in the drug market against different diseases and especially, orphan diseases. Organic extracts will be mentioned, but the rest are all aqueous.

### 5.1. Oxidative stress

#### 5.1.1. Oxidation

A study was conducted by Brito *et al.* [70] to evaluate the effect of *A. platensis* on preventing the oxidative stress condition generated from muscle damage and inflammation during exercise. This experiment was done on four rat model groups (each group has 2 rats): 1) trained rat without supplementation (control), 2) trained rat with 50 mg/kg of *A. platensis* surplus, 3) trained rat with 150 mg/kg of *A. platensis* surplus and lastly 4) trained rat with 500 mg/kg of *A. platensis* surplus. The rats were all set for 8 weeks training programme to strengthen them and all chosen rats’ weights were between 250 g and 300 g. The results were all compared with the control group (trained but without supplementation intake).

The main focus of this investigation was on three factors: measuring the 1) reactive oxygen species (ROS) by monitoring the malondialdehyde (MDA) and thiobarbituric acid reactive substances (TBARS) peroxidation by-products concentrations, 2) inflammation by monitoring the C-reactive protein (CRP) concentrations into three different areas; the blood plasma, liver and quadriceps, and lastly 3) muscle damage by monitoring the creatine kinase (CK) and lactated dehydrogenase (LDH) concentrations. The results were promising and represented a significant depletion of the CRP, MDA, TBARS, CK and LDH concentrations; therefore, those results would lead to ceasing inflammations in the systemic circulation. On top of this, the attenuation of the mentioned factors would contribute to prolonging the exercise period alongside healthy gains due to the depletion of CK and LDH concentrations. It is highly recommended that the same experiment to be applied to human athletes, including control trials.

Biliproteins present in *A. platensis* has a significant C-PC amount that exerts anti-oxidant and radical haunting properties. In addition, C-PC is a selective inhibitor of cyclooxygenase-2 (COX-2) and promotes apoptosis in lipopolysaccharide-stimulated Raw 264.7 macrophages (the Raw 264.7 cells are monocyte/macrophages-like cells [71]) [34], which have shown a tremendous pharmacological effect in treating colorectal cancer [23]. Besides, C-PC has shown a high similarity to chemical structures synthesized by the body naturally, such as biliverdin [23,72], and porphyrin, which is from a medicinal chemistry point of view, both possess potent anti-oxidant properties to scavenge reactive oxygen and nitrogen species free radicals. Besides, porphyrin acts as a chelating agent to reduce metal toxicity or poison. In addition, *A. platensis* is rich in phenolic acid, which exerts anti-oxidant activity by haunting hydroxyl radical and many other organic radicals that act as chain-breaking anti-oxidants, superoxide radical anion, and reducing agents. Furthermore, phenolic acids are essential compounds for changing pathways of cell signaling [73].

##### 5.1.1.2. Diabetes

The phycobiliproteins peptides in *A. platensis* were tested on type 2 diabetic (T2D) induced mice to figure out the role of the phycocyanobilin (PBP) in regulating the glucose level. It was witnessed and tested by Li *et al.* [74] that PBP has presented a dose–response graph and IC_50_ (Inhibitory concentration by 50%) between 0.5 and 1.0 mg/mL *in vitro* on dipeptidyl peptidase-4 (DPP-4) enzyme, which was tested on two different cultures: 1) *in situ* Caco-2 cells due to its high expression level of DPP-4, and 2) *in vitro* purified recombinant DPP-4. The results were compared with 1.0 µM of sitagliptin (reference/control group) to verify its pharmacological effect. Before the cells were used in this investigation, the author excluded any potential cytotoxic effects from the used models by performing 3-(4,5-dimethylthiazol-2-yl)-2,5-diphenyl-2H-tetrazolium bromide (MTT) assay to make sure that the chosen models used were safe, not cytotoxicated and viable before use to reduce bias and then it will be used after to record the inhibition percentage of the opted cultured cells after treatment.

The results were interesting since PBP concentrations falling in between 0.5 and 1.0 mg/mL against *in vitro* purified recombinant DPP-4 have hindered the cell viability in higher percentage (0.5 mg/mL of PBP; 40.5% ± 7.6%, 1.0 mg/mL of PBP; 62.1% ± 1.3% (VS) 1.0 µM of sitagliptin 79.5% ± 2.5%) compared to in situ Caco-2 cells (0.5 mg/mL of PBP; 16.4% ± 7.4%, 1.0 mg/mL of PBP; 29.2% ± 1.3% (VS) 1.0 µM of sitagliptin 89.6% ± 0.9%). The *in situ* results are strange compared to *in vitro* results despite both being incubated at the same time (30 mins) and temperature (37°C). The higher the concentration of PBP the better the results, but it will not be safe. The experimental results prove PBP similar pharmacological effect as sitagliptin; however, an *in vivo* experiment especially a human model experiment is required for further assessment.

DPP-4 is known for its significant contribution in regulating the incretins release such as glucagon-like peptide-1 (GLP-1) and gastric inhibitory polypeptide (GIP) mediated via G protein-coupled receptors present on β-cells’ surface plasma membrane. GLP-1 and GIP are responsible for stimulating the release of insulin to lower the blood glucose level. However, it was presented in a paper by Dupre *et al.* [75] and Seino *et al.* [76] that high levels of GIP inhibit the lipolysis and stimulate lipogenesis, eventually leading to obesity. The idea of inhibiting DPP-4 will help in not disturbing the incretins by lowering the blood glucose level. On top of this, *A. platensis* is rich in tocopherol (vitamin E), which plays a vital role in postponing the onset of diabetic drawbacks and hindering the progression of diabetic complications [77]. Vitamin E deficiency is detected by the disturbance of tocopherol serum concentrations, except in patients with weak lipid absorption [78].

Besides, a meta-analysis study was done by Hatami *et al.* [79] on eight studies to test the effect of *A. platensis* on fasting blood glucose. The study found a significant reduction in fasting blood glucose of almost (–) 17.88 mg/dL with a 95% confidence limit. Furthermore, the author mentioned that there was no significant effect on hemoglobin A1c (HbA1C) or post prandial blood sugar after *A. platensis* consumption.

##### 5.1.1.3. Inflammation and immunomodulation

###### 5.1.1.3.1. Interleukins

A systemic review and meta-analysis study was conducted on 11 articles (from 2005 to 2018) after applying the inclusion and exclusion criteria, including identity, screening, eligibility and extracting relevant trial studies, including controls done by Mohiti *et al.* [80] on the significance of *A. platensis* in affecting oxidative stress (MDA and TBARS) and pro-inflammatory cytokines (Interleukin-2 (IL-2), Interleukin-6 (IL-6) and Tumor Necrosis Factor-alpha (TNF-α)). Overall, all the data presented in the 11 studies included participants of genders, irrelevant ages and races, and different body mass indexes (BMIs). The dose range falls between 1 and 8 g/day, and the data were all collected using Preferred Reporting Items for Systematic Reviews and Meta-Analyses (PRISMA) and analyzed using Statistics (STATA) and ANOVA Software. The heterogeneity was assessed using Cochrane’s Q test at p <0.05 significance level, and I-squared statistics were also involved. On top of this, the author used Begg’s and Egger’s tests to asses the small-study effects to reduce bias.

All the results achieved a 95% confidence limit with significance [80], admitting that *A. platensis* supplementation boosted IL-2 concentrations substantially but had no effect on TNF-α or MDA levels. However, with the participation of both genders, the MDA levels have fallen significantly. *A. platensis* consumption had little influence on IL-6 and TBARS levels. According to the analysis findings from subgroups, the *A. platensis* supplementation resulted in a substantial decrease in IL-6 and TBARS concentrations when the baseline BMI of participants was less than 25 kg/m^2^. Furthermore, when both genders participated in studies, MDA levels fell significantly. The physiological outcome of lowering IL-6 leads to the depletion of fever and inflammation by decreasing the synthesis of CRP [81], whilst lowering TBARS and MDA means that less exciting free radicals are emitted from lipid peroxidation due to the low reactivity, which will avoid inducing or exacerbating the oxidative stress condition around its region [82].

###### 5.1.1.3.2 Allergies

*A. platensis* modulates the immune system functions, for instance suppressing mast cells from secreting histamine by inducing anti-inflammatory properties [83]. According to Mao *et al.* [84], there was a reduction of 32% in interleukin-4 (IL-4) levels after a patient consumed a high dose of *A. platensis*, in addition, it scientifically showed the defensive effect of this microalga against substantial allergic rhinitis reaction [11]. *A*. p*latensis* can modulate the immune system by replacing nutrient deficiencies since the deficiency of nutrients is related to changes in immunity, which behaves by changing the T-cells’ development, secretory IgA antibody response, cytokines, and natural killer cell (NK-cell) activities [34].

##### 5.1.1.4. Cancer

The effects of the immune regulation associated with anti-oxidants have been contested that *A. platensis* has a possible tumor-destroying (anti-tumor) mechanism and hence has a role in preventing cancer [85]. The therapeutic function of *A. platensis* was clinically developed previously on hamsters, whereby it demonstrated a tumor recovery after topical application [20]. Besides, the enteral application of *A. platensis* extracts for oral carcinogenesis, in particular leukoplakia, which was clinically proven that the therapeutic function of the extract has a promising cure for leukoplakia and other diseases [85–87].

*A. platensis* major bioactive metabolites, especially alkaloids, had significantly inhibited the hepatocellular carcinoma cells *in vitro* with an IC_50_ of 0.53 ± 0.08 mg/mL. Almost 80% of cell viability had been reduced [88]. The author mentioned that the remedy concentration was chosen based on the dose–response trend and MTT assay (after 24 hrs of treatment) results to prove that the chosen alkaloid IC_50_ is safe and not cytotoxic on normal cells. The alkaloid was chosen after it was experimentally compared with other bioactive molecules from water and methanol extracts, representing that *A. platensis* derived alkaloids are stronger anticancer drugs than other molecules from *A. platensis*.

For further proof, more investigations have been reported with an objective of extracting, isolating and purifying alkaloids and phenolic compounds from *A. platensis* [89–91] against particular cell lines, for instance, hepatoblastoma (HepG2 cells) (IC_50_ 22.3 µg/mL of *A. platensis* aqueous extract) [92], colon carcinoma (HCT116) (IC_50_ 18.8 µg/mL of *A. platensis* aqueous extract) [92], acute leukemia (Kasumi-1) (IC_50_ 3.68 µg/mL of *A. platensis* ethanol extract), chronic leukemia (K562) (IC_50_ 4.64 µg/mL of *A. platensis* ethanol extract) [93], pancreatic cancer cells (IC_50_ 60 µM of PCB water extract and IC_50_ 125 µM of chlorophyllin water extract)) [94], human melanoma (A375) (IC_50_ 40 µM of Selenium-Phycocyanin (Se-PC) aqueous extract), human breast adenocarcinoma (Mcf-7) (IC_50_ 40 µM of Selenium-Phycocyanin (Se-PC) water extract), and human fibroblast (Hs68) (IC_50_ 40 µM of Se-PC water extract) [95]. All the extract’s IC_50_ were compared with other extract solutions, including control groups. The selection criterion was based on the lowest IC_50_ concentration that could achieve the highest inhibition percentage on the viability of the infected cultured cell lines detected from dose–response trends.

##### 5.1.1.5. Cholesterol and lipoproteins

According to Mani *et al.* [30,34], a clinical study was performed on 15 diabetic patients who were given *A. platensis*. As a result, a significant depletion in high-density lipoprotein was witnessed that affected the low-density lipoprotein (LDL: HDL) ratio. This research, though limited, indicates that it can be accepted as a drug in the long term. Further evidence was observed in patients with HDL levels that were significantly increased, with no significant reduction in LDL cholesterol after 8 weeks of therapy; however, the atherogenic effect also decreased considerably [96]. A lipid profile has been produced after the patients have ingested *A. platensis* supplementation for testation, and a favorable response was observed. One of the essential fatty acids is gamma-linolenic acid (γ-LFA), which prevents fat accumulation. *A. platensis* composition contains appreciable amounts of γ-LFA, which could reduce serum cholesterol levels; this could be attributed because of the high fiber and protein content of *A. platensis*; therefore, decreased VLDL triglyceride production and increased VLDL disappearance in the periphery [30].

An experimental study was done by Serban *et al.* [97] on alloxan-injured animals, whereby resulted that phycocyanin reduced total cholesterol (TC) and total glyceride (TG) levels in the blood, raised hepatic glycogen levels, and preserved glucokinase expression in the liver. This investigation was backed up by a meta-analysis study, which included 7 clinical trials to examine the effect of *A. platensis* supplementation on plasma lipid concentrations. All the mentioned results have achieved a 95% confidence limit of significance on the following factors: low-density lipoprotein cholesterol (LDL-C), which was reduced by (–) 1.1 mmol/L, TC by (+) 1.2 mmol/L, and TG by (–) 0.5 mmol/L [98].

However, an increase was witnessed in high-density lipoprotein cholesterol (HDL-C) by (+) 0.16 mmol/L, which is an interesting discovery to known lipid-lowering effects of *A. platensis*, but till now, the mechanism of action and pathway is unclear [99]. Another meta-analysis study was done by Hatami *et al.* [79] on 8 studies to test the effect of *A. platensis* on lipid profile. All the studies’ results achieved a 95% confidence limit with significance. The study found a significant reduction in TG of almost (−) 30.99 mg/dL, TC of almost (−) 18.47 mg/dl, LDL-C of almost (−) 20.04 mg/dL, and very-low-density lipoprotein (VLDL) of almost (−) 6.96 mg/dL. However, a significant increase in HDL-C of almost (−) 6.96 mg/dL was witnessed. This concludes that *A. platensis* have a positive influence scientifically on blood lipid profiles, which will decrease the susceptibility of mankind from developing, for instance, coronary heart disease, stroke, or cardiovascular diseases [100]. This definitely has to be critically monitored and aided with well-balanced nutrition, regular sports, optimum water intake and a general healthy lifestyle to create a suitable environment for the extract to work properly.

##### 5.1.1.6. Hypertension

In a study conducted by Pan *et al.* [101] to investigate 10 mg/kg of *A. platensis* hydrolyzates and isolated tripeptides such as Ile-Gln-Pro (IQP) and Val-Glu-Pro (VEP) against hypertensive rats was conducted to verify their effect on the renin-angiotensin system (RAS) in the myocardium to treat hypertension. After 6 weeks of treatment and 2 weeks of observation, the author witnessed the blood pressure decrease and was assured further after observing the mRNA expression levels and remedy concentrations to assure if they were affected or not affected. The results were compared with distilled water (control group), which resulted in a significant anti-hypertensive effect. However, the author reported that angiotensin-converting enzyme (converts angiotensin I to II to increase blood pressure) [102], angiotensin II (Vasoconstricts vessels) [103] and angiotensin type 1 receptor (Increases aldosterone, which means high sodium reabsorption/turnover) were attenuated. However, the angiotensin type 2 receptor (Increases cGMP levels, which leads to vasodilation) [104], Mas receptor (Interacts with angiotensin-(1-7) and induces an inhibitory effect against inflammatory cytokines) and angiotensin-(1-7) (Vasodilator agent) were activated [105]. In addition, another study has found a peptide derived from *A. platensis* called SP6 (GIVAGDVTPI) found to be promising against atherosclerotic and hypertensive animal models. The research group finds this as a green light for further investigation to prove it in preventing and treating multifactorial cardiovascular diseases [106]. According to Carrizzo *et al.* [107], ‘SP6 (GIVAGDVTPI) exerted direct endothelium-dependent vasodilation of *ex vivo* vessels, an effect occurring via a PI3K (phosphoinositide-3-kinase)/AKT (serine/threonine kinase Akt) pathway converging on NO release’.

##### 5.1.1.7. SARS-CoV-2/COVID-19

Since COVID-19 is the most controversial topic up-to-date and a matter that deserves attention, this review compiled some promising literature reviews insights on the role of *A. platensis* against SARS-CoV-2/COVID-19. There are *in silico* and *in vitro* studies published today discussing the promising inhibitory effect of Spirulina’s metabolites against the proteases of SARS-CoV-2/COVID-19 and its variants. Interesting data have been gathered for clinical scientists/virologists to refer to consider natural compounds demonstrated as an effective remedy against viruses. Promising results will be achieved if those compounds are applied against SARS-CoV-2/COVID-19.

A molecular docking study has been performed on various natural metabolites, and one of them was Spirulina’s phycobilins products such as PCB tetrapyrrole chromophore and many more. According to Pendyala [108], the docking generated on the main protease ligand (M_pro_-ligand) has resulted in being bounding with a binding energy that did cut off at −7.6 Kcal/mol. Another trial was on papin-like protease ligand (PL_pro_-ligand), which resulted in binding energy cutoff at −8.0 Kcal/mol accompanying a higher binding affinity compared to Mpro, but still, both of them are relatively effective. Another study by Pendyala [109], reported that PCB showed a potent inhibition activity compared to other compounds, with an IC_50_ value of 62 μM. The *in silico* docking and *in vitro* enzyme inhibitor promising results gave insights on PCB to be utilized as an effective, potent inhibitor against SARS-CoV-2 M_pro_ and PL_pro_ endogenous proteins.

Another similar study has been investigated by Pendyala [108], but on a different site called RNA-dependent RNA-polymerase (RdRp), which resulted in that PCB demonstrating a superior binding affinity score in comparison to most of the screened anti-viral drugs. PCB developed binding energy of −9.3 Kcal/mol. In comparison to Lopinavir (−9.7 Kcal/mol) followed by Nelfinavir (−9.3 Kcal/mol), and lastly Remdesivir (−9.0 Kcal/mol). The phytochemical bioactive natural drugs used were descendingly ordered in the following order: Riboflavin, Cyanidin, Daidzein, and Genistein whereby demonstrated comparable binding affinity that makes them potential candidates against SARS-CoV-2/COVID-19 for pharmaceutical companies to consider and develop further verification and validation assessments by performing an *in vitro* study [108,110].

Pendyala (DOI: 10.26434/chemrxiv.12051927) shared an experimental *in silico* pre-print of molecular docking results using PCB extracted from *A. platensis* against protease SARS-CoV-1/COVID-19. The optimum binding affinity for PCB reached −7.2 kcal/mol and was evaluated further using the AdmetSAR2 prediction tool to predict drug toxicity. Whereby it resulted with no evident unfavorable strength. However, PCB developed two inhibitions against CYP1A2 and CYP2C9 with a probability of 0.52. To the best author’s knowledge, there is no significant carcinogenicity or mutagenicity or drug interaction was observed from administered high LD_50_ (lethal dose by 50%) (500 and 5000 mg/kg). Phycoerythrobilin (PEB) shows an interesting binding affinity of −7.3 kcal/mol. The PEB biomolecule demonstrated no potential inhibition of the following cytochromes: CYP1A2, CYP2C19, CYP2C9, CYP2D6, and CYP3A4. Since PEB presents more negative binding energy than PCB and no drug interaction was obvious, it is highly recommended to opt for a safer compound than being more effective because patient safety is always a priority. In this situation, it is preferably PEB compound to be applied *in vitro* and *in vivo*.

Petit *et al.* [111] recommended Pendyala pre-print work and introduced the same idea, but to be applied on SARS-CoV-2/COVID-19 by screening 48 components, including PEP, PCB and Phycourobilin against SARS-CoV-2/COVID-19. The molecular docking results from particular compounds were promising and possessed a strong antiviral activity with strong binding energy. The molecular docking results were tested on two different docking tools; Autodock Vina and SwissDock Softwares. The up-to-date corrected results by Petit *et al.* [112] are in the following: PEB (−7.45 ± 0.05 kcal.mol^−1^ Autodock Vina (VS) −10.35 ± 0.00 kcal.mol^−1^ SwissDock) PCB (−7.25 ± 0.15 kcal.mol^−1^ Autodock Vina (VS) – 9.35 ± 0.025 kcal.mol^−1^ SwissDock), and lastly Phycourobilin (−7.1 ± 0.00 kcal.mol^−1^ Autodock Vina (VS) −9.285 ± 0.425 kcal.mol^−1^ SwissDock). The author assessed their toxicity using an *in silico* toxicity analysis tool and reported that all the mentioned compounds are safe and have high oral bioavailability.

Phycocyanin derived from *A. platensis* is structurally similar to biliverdin, which means that it possesses a similar anti-oxidant effect [113] and similar pathway metabolism via biliverdin reductase [114]. Phycocyanin inhibits the NADPH oxidases, which makes it challenging to generate and activate reactive oxygen species, hence eventually oxidative stress conditions due to the non-stoppable chain reactions, which are correlated to the increase of inflammatory signals. According to McCarty [115], McCarty *et al.* [116], and Zheng *et al.* [114], the NADPH oxidases inhibition would stop the inducement of further inflammation due to the hindrance and control of ROS and reactive nitrogen species (RNS). As was mentioned by McCarty [115] and McCarty *et al.* [116] that this inhibition is a promising pathway for eradicating myriads of viruses.

Several randomized controlled trials (RCTs), systematic reviews, and possible clinical trials have been implemented to investigate the efficacy of *A. platensis* in treating several diseases, which clinically was proven *in vivo* and *in vitro* that this alga may enhance several symptoms and have an antiallergic, anticancer, and anti-viral effects [34,83]. Moreover, anemia, hepatotoxicity, dyslipidaemia, immunodeficiency, inflammatory processes, and cardiovascular diseases [29]. In addition, Spirulina extracts have been reported to possess anti-inflammatory properties since their mechanism as an anti-inflammatory agent has been demonstrated successfully against inflammations related to beta-coronavirus infections in the lower respiratory region of the lungs. However, there were no other studies against different variants [117,118].

The advantage of taking herbs and algal products has been shown clinically to have anti-viral properties that can be used for immunomodulation and existing infection [34,83]. According to Chen *et al.* [38], clinical trials have shown that *A. platensis* extract reduced viral replication in cell cultures. It inhibited the formation of viral plaque in a broad range of influenza strains *in vitro*. It was regarded as safe and well-tolerated at high doses of *A*. platensis extract in cellular and animal toxicity studies. The strong demand for anti-viral action was indicated by the impact of calcium ion chelation on molecular conformation in groups of sulfates [52].

##### 5.1.1.8. Microbes

Growth inhibition of some drug-resistant bacteria such as *Escherichia coli, Klebsiella pneumoniae, Pseudomonas aeruginosa*, and *Staphylococcus aureus* was significantly evident in the culture after purifying and applying C-PC from *A. platensis* on them, and hence demonstrated significant anti-bacterial action on six strains of Vibrio [40,119] due to the presence of γ-LFA, which has an anti-microbial activity function [63]. *A. platensis* has also exhibited anti-fungal activities discovered by Gorobets *et al.* [120] when applied to the culture fluid. The variant of *A. platensis* doses has had significant stimulating and inhibitory effects in cultivated microorganisms because of the various metabolites involved in the nutrient agar preparations. Besides, *A. platensis* was reported to enhance and increase the lactobacillus bacteria’s health in the intestine, which enabled vitamin B6 to be absorbed efficiently and induced the release of energy [34,83].

A disc diffusion microbial susceptibility testing method [121] was used by El-Baz *et al.* [122] to study the anti-microbial effect of *A. platensis* extract solutions against *Escherichia coli, Salmonella typhi, Staphylococcus aureus, Enterococcus faecalis*, and *Candida albicans*. Despite the fact that there were no inhibition zones against *Escherichia coli* and *Salmonella typhi* (Gram-negative bacteria and belongs to the Enterobacteriaceae) and *Staphylococcus aureus* (Gram positive bacteria and belongs to the Firmicutes phylum), obvious inhibition zones were witnessed only against *Enterococcus faecalis* and *Candida albicans* (Diploid fungus) [123] in the presence of *A. platensis* ethanol extract. The zones with negative zones of inhibitions means that they were not affected by the ethanol extract of *A. platensis*.

## 6. Physico-chemical properties

According to Abdel-mawla [35], after the *A. platensis* powder sample was fed into a dryer calibrated with a specialized atomizer diameter pore. It, later on, influenced the trichomes’ initial size and affected the particle size distribution of the *A. platensis* sample. The physical properties of *A. platensis* (g/100g sample, on a dry weight basis) were studied and have determined the bulk density (0.84 ± 0.02 Kg/lit) of *A. platensis* product after it was influenced and analyzed the particle size distribution (100% mesh), appearance (Fine, uniform powder), color (Blue-green (cyano) to green), odor taste (Mild like seaweed) and lastly its consistency form (Powder form). *A. platensis* is considered as an alkaline food because of its identified pH which helps in promoting alkalinity in the body; this, in turn, promotes increased bone mass since the body is not required to sacrifice calcium to balance its pH, hence found to enhance metabolic functions efficiently [124].

Zhong *et al.* [125] investigated *A. platensis* release and degradation *in vivo*, which was loaded with an anti-cancer drug named doxorubicin (DOX). The drug release kinetics of this combination was assayed in different pH values of buffer solutions ranging from 5.5 to 9.0, to validate the value of the buffer’s pH with the most appropriate and efficient release. The results showed a pH-responsive release of DOX from *A. platensis* carrier with a considerably enhanced release at lower pH values, with an observed 72 h time-dependent release with around 62% release at pH 5.5 and more than 58% release at pH 6.5. The lower the pH, the better the releases and hence makes *A. platensis* behavior a promising agent for drug release of its constituents and contents. One of its compositions is C-phycocyanin which proves a significant anti-cancer and anti-tumor properties that could be administered intravenously, whereby the constituents would be released the moment it is exposed to tumors because of the acidic characterization of the tumor microenvironment [39,126].

Medical researchers reported on various diseases behaviors and were revised by myriads scientists that all agreed with the medical researchers’ hypothesis that widespread diseases, for instance, cancer, has more chance to be prone to if the host provides an acidic environment [127]. Regular use of *A. platensis* could help keep the body alkaline which would eventually lead to helping to reduce the risk of lowering the body’s pH and is known to be the ideal food supplement for reducing and balancing a patient’s weight. The body must be close to low alkaline pH to reduce the risk and susceptibility, which is stated to remain between pH 7.35-7.45 ranges via respiratory and metabolism homeostasis, including the human diet [124]. This is one of the main functions of *A. platensis*, which is highly recommended by a nutritionist to be used as a nutritional supplement [128]. The microbiological quality of *A. platensis* has been tested and resulted in no detection of viable bacterial count and mesophilic spore formers bacteria [35].

## 7. Spirulina culturingagricultural prospects

The optimum temperature for Spirulina *sp*. growth lies in the range of 30°C to 35°C. According to Oliveria *et al.* [129], the best result has shown that the optimum temperature is 30°C. In contrast, biomass production resulted in 68.67% (*A. maxima*) and 64.35% (*A. platensis*), as illustrated in Table 2. These high protein levels indicate the commercial availability of the tested Spirulina *sp*. species as food products in aquaculture. Furthermore, Belay [49] revealed that *A. platensis* accumulated large amounts of g-linolenic acid (GLA) compared to *A. maxima*.

**Table 2.** Comparison between *A. maxima* and *A. platensis* on the effect of temperature on the mean composition of the final biomass in 4 L fermenter.

*Arthropsira* species	*Arthrospira maxima*	*Arthrospira platensis*
Temperature °C	30°C (Optimum)	30°C (Optimum)
Proteins (% DW)	68.67 ±0.68	64.35 ±1.24
Carbohydrates (% DW)	64.35 ±1.24	64.35 ±1.24
Lipids (% DW)	6.20 ±0.50	6.96 ±0.86

DW; Dry weight %

Most production companies use *A. platensis* instead of *A. maxima* as their active pharmaceutical ingredient (API) which is regarded as a dietary health supplement. This is because *A. platensis* has more carbohydrates and lipids in its composition profile, as illustrated in Table 2 [129], which was opted more by scientists and recognized as a healthcare supplement to humankind [14,46,129]. However, this does not mean that *A. maxima* are not used; other companies use it. The point of **[130,131]discussion here depends on the regional area that can accommodate the most suitable species because the environment is their home and every species opt for their favorable zone to grow.

## 8. Targeted-CRC drug delivery system

Since C-PC is the most abundant constituent in *A. platensis* aqueous extract, which throws great attention to scientists to consider C-PC for RCTs to prove to the FDA the potential use of Spirulina as an anti-cancer therapeutic compound against colorectal cancer (CRC). This area was chosen because of *A. platensis* aqueous extract including C-PC’s pH/pKa, which are close to the intestine environment’s pH/pKa for better bioavailability.

The promising news is that *A. platensis* is usually neutral and close to alkaline. However, the intestine is alkaline; hence *A. platensis* increases its alkalinity further after reaction and makes the intestine more alkaline than it already is, which does not fit the colon or rectal cancer favorable acidic environment and eventually inhibits its activity. The ease of drug or supplement absorption to enable an active therapeutic effect is termed bioavailability; the higher the drug or supplement bioavailability, the better its effect. According to Sotiroudis *et al.* ^130^, there are various studies have been performed on the characteristics and bio-composition of Spirulina as natural food *in vitro* and had resulted in high energy in return from both low-calorie carbohydrates and fat aided with high digestibility. According to Stanic-Vucinic *et al.* [41], pepsin localized in simulated gastric fluids digested C-PC quickly. After pepsin degradation of Spirulina’s C-PC constituent, resulted in a size change of around 2–13 amino acid residues of its chromopeptides that were found to induce a promising metal-chelating property and anti-oxidant activity aided with potential cytotoxic effect on cancer cell lines. C-PC could be tried against CRC.

According to Stanic-Vucinic *et al.* [41], their research team has witnessed several studies applied *in vitro* on C-PC in cell culture. The team observed the location of chromopeptides protein inside the cells, whereby drug delivery scientists would have an idea to use specific isolating methodologies to approach it. However, it remains not clear to vividly locate the protein, which made it difficult to encapsulate C-PC with protein carriers to penetrate cells by delivering both Spirulina as a whole or C-PC and APC solely as active pharmaceutical ingredients (APIs) using specific dosage forms with the new drug delivery techniques applied.

It is highly recommended to leverage *A. platensis* consumption by providing further preformulation studies that deliver *A. platensis* either using *A. platensis* as a whole or extracted CPC and APC constituents as an API using the discussed potential dosage forms, tablets or intramuscular (IM). More emphasis is on producing a better quality and efficacy in the delivery methodology of the aqueous extract in tablets and IM dosage forms.

The hypothesis used in IM dosage form is to increase the efficacy of *A. platensis* by delivering it in stealth femtoliposomes from synthesized lipids or natural lipids or via bioengineering blood cells [132] for advanced drug delivery. This is because the cells will have better bioavailability, absorption, distribution, less interaction within the body micro-components, in other words, biocompatible, and extend the release of *A. platensis* aqueous extract. This requires increasing the number of carries and reducing each carrier’s dose, as further explained in Section 7. The hypothesis used in the tablet dosage form is to deliver *A. platensis* with modified polymers to delay-release (DR) the tablet core active ingredient. To have a more effective delay than it is already and to prevent premature drug release of constituents in the stomach aided with a secondary coating layer with a sustained-release (SR) polymer to release the active ingredient throughout the intestine and to promote the absorption of *A. platensis* constituents better in the intestine by reducing polymer size to femtoscale, as further explained in Section 8.

## 9. Parenteral femtoliposome formulation

### 9.1. Intramuscular parenteral dosage forms

Using*A. platensis* extract in IM parenteral dosage form for the first time is chosen based on the procedural algorithms for IM injections and formulation of set guidelines, which are essential in assuring effective pharmacokinetics and pharmacodynamics profiles of the drug. Also, the IM route is less risky compared to IV. Despite the IV route mechanism eliminates the other metabolism impacts, it allows the substance fractions to bypass the first-pass metabolism for better permeation to the systemic circulation site [133].

However, it is not risky compared to SC and ID dosage forms. IM has higher efficacy as it is nearer to the bloodstream and the assimilation and diffusion processes are faster than other parenteral dosage forms. In addition, IM is less risky and more effective with almost 100% drug bioavailability, effectively targeting the drug to the primary target site faster than SC and ID dosage forms. Therefore, the IM route is commonly indicated for patients who are non-compliant, uncooperative, reluctant, and unable to receive drugs through other commonly utilized routes [133,134]. The ventrogluteal site is considered the safest for IM injection due to the thin plane of subcutaneous tissues and the relatively thick bulk of the underlying muscle [133,135]. Each category has its injection site based on the muscle maturity, and intake of volume, such as infants’ site of injection is vastus lateralis, children’s site of injection is vastus lateralis and deltoid. Adults’ site of injection is ventrogluteal and deltoid [134], as illustrated in Table 3 [133,134].

**Table 3.** The common injection sites used for IM parenteral dosage form with valid recommended volume and patient positioning before administration.

Injection site	Recommended volume	Patient positioning
Deltoid	1.0-2.0 mL	The arm located on the waist to relax the muscle while standing or sitting
Ventrogluteal	2.5-3.0 mL	The prone position facing away while lying on the sideways
Rectus femoris and vastus lateralis	Up to 5.0 mL	The toes pointed away to relax the muscle while lying or sitting

### 9.2. Opted parenteral dosage form

The ease of operation, adherence, therapeutic target, dosage form, and toxicological properties and effects are done based on selecting the route before implementation [136]. In other terms, the product is improperly absorbed and undergoes minimal bioavailability if the formulation is not compatible and administered correctly.

The IM injection drugs diffuse rapidly into the systemic circulation because of the rich vasculature of the striated muscle. Water-soluble drugs diffuse faster, whereas an oil-based vehicle with dissolved substances disperses the drug more gradually. The avoidance of the liver’s first-pass effect promotes bioavailability along the IM route of administration to 100%. According to Vidya *et al.* [53], *A. platensis* aqueous extract has both properties, water-soluble fraction that was discovered to reduce the amount of serum glucose while fasting effectively; hence, water-insoluble fraction oppressed glucose levels during a glucose load. Also, it reduced cholesterol, triglycerides, and LDL cholesterol in the blood. As a result, it acted as an anti-hyperlipidemic agent in clinical reports [14,16,29].

Thus, this made it challenging to be able to conceive an IM injection for *A. platensis*. However, it is possible to introduce the stealth femtoliposome vesicles coating idea to make it conceive and deliver *A. platensis* through IM, although it has both water-soluble and water-insoluble properties due to its biochemical composition. This ingredient has been scientifically proven to have much valuable feedback if it is implemented since *A. platensis* scientifically proved its significant therapeutic functions by proactively implementing it. The new drug or supplement delivery technique makes it possible to deliver constituents at a femtoscale by increasing the number of vesicles/carriers and decreasing both their size and dose per vesicle/carrier for better flowability, distribution, and less interaction with any interventions. This concept reduces drug-drug interaction and increases drug–disease interaction since the size difference is major, which grabbed the attention of improvising this new delivery technique and applying it with *A. platensis* for the first time.

The IM route dosage calibration is restricted by the small volume of administration, which is considered according to Bolger *et al.* [136], preclinical animal species (0.05–0.5 mL/kg) and human (2–5 mL/kg). Furthermore, the pace of administration should be gradual, and the dosage should not be painful to minimize muscle injury, nearly 10 sec/ml [133], and the drug formulation should be nonirritating [136,137]. Therefore, the IM route option has been chosen for several factors, as illustrated in Table 4 [133,134].

**Table 4.** Comparison between the advantages and disadvantages of intramuscular (IM) injection route and other related parenteral dosage forms.

Intramuscular (IM) Injection Route	
Advantages	Disadvantages
A faster rate of absorption compared to subcutaneous (SC) and intradermal (ID) injections	A slower rate of absorption compared to intravenous (IV) injection. However, risky compared to IV injections
Capable of absorbing larger volumes of solutions compared to SC, IV, and ID injections	General discomfort is expected, such as a painful sensation at the site of injection
The drug diffuses into the systematic circulation rapidly	Tingling and numbness sensations
Almost 100% bioavailability	Sometimes causes swollenness at the site of injection
It also avoids the gastric factors governing the drug absorption	The rate of administration at the site of injection will be slow to avoid injury to muscles

### 9.3. Liposomes classifications

**Figure 3.**
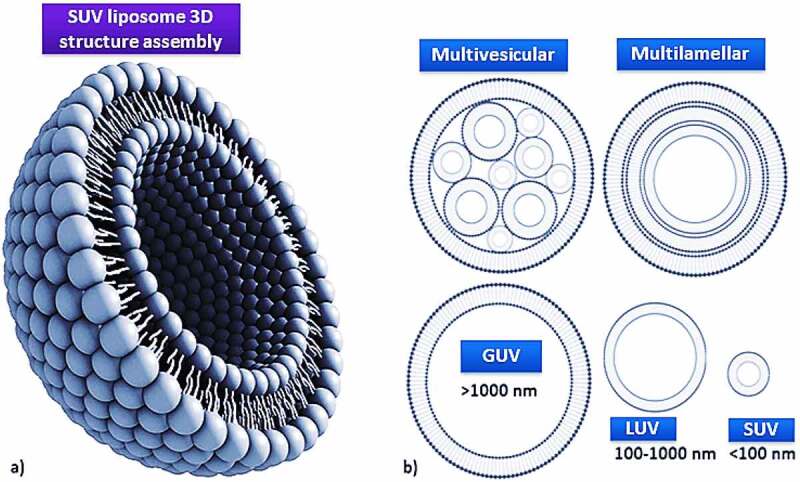
Liposome assembly representation of phospholipids in a bilayer (a) aided with various lamellar forms and sizes (b).

**Figure 4.**
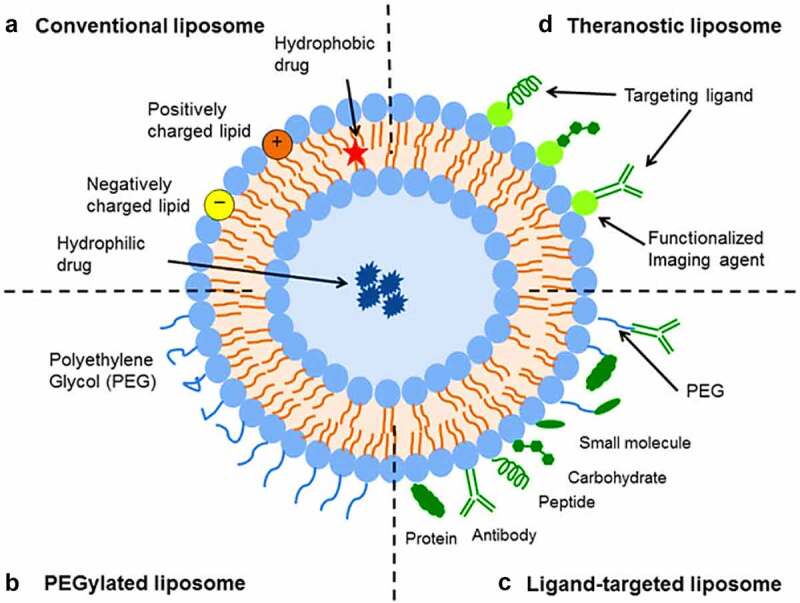
SUV liposome carrier in different formulations.

Liposomes are biodegradable, amenable, and biocompatible to merge both hydrophilic and lipophilic drugs [138], making them an efficient drug delivery system composed of double-layered phospholipids. There are various formulations of liposomes, including multilamellar vesicle (MLV) that consists of several lamellar phase lipid bilayers, the small unilamellar liposome vesicle (SUV) that consists of one lipid bilayer, the large unilamellar vesicle (LUV), giant unilamellar vesicle (GUV). Both the LUV and GUV consist of one lipid bilayer but are sizable, and the cochleate vesicle; a product produced from the fusion of unilamellar lipid vesicles, multivesicular; that consists of large unilamellar vesicles filled with small unilamellar vesicles encapsulated inside [139–141], as illustrated in Figure 3 [142,143]. On top of this, there are many features expressed by liposomes such as conventional, PEGylated, ligand-targeted, and theranostic liposomes for drugs or supplements to be delivered in, as illustrated in Figure 4 [144]. A pH-dependent polymer is recommended to coat the surface of liposomes to avoid the destabilization of liposomes under acidic conditions. It could be further specialized by adding ligands to enhance the site-specificity [139].

An intrinsic feature phenotype that emerged from tumors has been discovered to distinguish this feature from other normal tissues, which is the extracellular pH (pH_e_) [145–147]. It opened doors for scientists to find solutions to tackle this issue by delivering drugs against cancerous cells in an effective way to make the drug uptake and activity suitable and achievable for these malignant tumors to be reduced and eventually cured. The lower pH_e_ experienced in the region is because cancerous cells reach to high metabolic rate and commence to proliferate and metastasis due to the high anaerobic glycolysis reaction that produced excessive lactic acid [146–150] and carbon dioxide [150] without efficiently being removed. According to Leeper *et al.* and Thistlethwaite *et al.* [145,150], the mean pH_e_ value was recorded from an experiment the authors conducted by using electrical and chemical probes fluid is approximately 7.06 with a range of 5.7–7.8 [150,151]. As a result, the tumors at low pH_e_, slow the uptake of weakly basic drugs due to high drug resistance, which is exploited as a drug-release trigger [152]. The low pH_e_ parameter makes it difficult to cure cancerous cells using non-chemotherapy drugs.

#### 9.3.1. Introduction to small unilamellar vesicle stealth femtoliposomes

One of the best and easiest methods for preparing SUV cationic and anionic liposomes is that some lipids dissolve in water readily. The method involves injecting a small volume of ethanolic lipid solution into a large volume of water resulting in a homogeneous mix of lipids that immediately dilutes ethanol in a high excess of water due to the injection force. According to Shen *et al.* [151], this method generated mainly liposome vesicles with diameters around 25–50 nm of small unilamellar vesicles (SUVs) and is recommended by the author to be suitable and adequate for cationic liposomes using ethanol injection methodology [153].

According to Deshpande *et al.* and Qi *et al.* [154,155], although the cationic liposomes tend to localize in newly formed tumor vessels, their positive charge on the surface results in nonspecific interactions with the anionic species in the blood. Therefore rapid clearance from circulation by the reticuloendothelial system (RES), which decreases the enhanced permeability and retention (EPR) effect. According to Danhier *et al.* [156], liposomes should typically be less than 400 nm in size to take advantage of the EPR effect. For extravasation into tumors, the vesicle threshold of about 400 nm has been recorded. However, it has become more efficient for many particles less than 200 nm to be extravasated. This made us to thought about inventing and introducing this new method to use and deliver medicines in SUV stealth femtoliposomes size range; to obtain better extravasation.

According to Egusquiaguirre and Torchilin *et al.* [93,157], various overexpressed receptors could be used to treat cancerous cells, for instance, folate and transferrin (Tf) receptors (TfR). Other biomarkers such as Arginine-Glycine-Aspartic Acid (RGD), anti-nucleosome antibodies, antibodies against vascular endothelial growth factor (VEGF), vascular cell adhesion molecule-1 (VCAM-1), matrix metalloproteases, and integrins. Delivery of drug-loaded liposomes directly to their organelle of action enhances the therapeutic window and reduces its adverse side effects; these biomarkers have been used to make liposomes tumor cell-specific.

### 9.4. Preparation of spirulina femtoliposome formulation

#### 9.4.1. Cationic femtoliposome formulation

Since cationic femtoliposomes are needed to be achieved, it requires further processing by adding the range size of 25–50 nm of liposomes filled with *A. platensis* in an evaporation thin-film apparatus and increasing the hydration time to reduce the size further below the range of 20–50 nm up till it reaches to femtoliposomes (10^−15^) size. Then, increase the flow rate (FR) from 2:1 to 5:1, which is stated by the nano-assembler that at high FR in turbulent mode, decreases the size and by setting it at 5:1; this should produce a very small size. According to Vemuri *et al.* [158], the usual volume of encapsulated volume (µl/mg lipid) in an SUV has a 20–100 nm size range from 20 to 100 µl/mg, which means that *A. platensis* extract inside the SUV will be from 20 to 100 µl/mg. Therefore, in femtoliposomes, the volume of *A. platensis* encapsulated will be from 5 to 20 µl/mg and covert it to femtoliter (10^−15^) since the liposome will be in femtosize.

The measurement of heterogeneity in a sample is dependent on the size of particles during analysis or isolation [159], whereby the size could be measured by the dynamic light scattering (DLS) technique utilizing a NanoZS Zetasizer to detect the Brownian motion frequency of particles that were utilized and recommended by Benne *et al.* [160] to measure the anionic liposomes in nanosize or smaller. Furthermore, the same instrument was used to measure zeta (ζ)-potential by laser Doppler electrophoresis [161], a technique for determining particles’ velocities by measuring the Doppler shift of laser light scattered from them.

The methodology utilized by Benne *et al.* [160] to measure anionic liposome size will be replicated using a different formula to measure cationic femtoliposomes size, whereby it will be diluted 100-fold in phosphate buffer (PB) to a total volume of 1 mL for these measurements. Particle concentration will be measured using nanoparticle tracking analysis (NTA) [162] or UV-Vis spectroscopy and apply it on cationic femtoliposomes for optimal atomic force microscope (AFM) measurements. The same experiment and methodology will be replicated to measure the size and diameter of femtoliposomes. Based on the DLS attenuation, the femtoliposomes will be diluted in PB to a particle concentration between 107 and 109 particles/mL. NTA measurements will be recorded using a NanoSight LM20.

According to Liu *et al.* [163], their team discovered a potential specific targeting ligand as the biomarker for colorectal cancer, which is CRC-9 which consists of natural, unnatural amino acids and small molecules. For *in vivo* application, the authors expected the potential for CRC-9 to resist proteolytic degradation and become stable. Thus, CRC-9 is a peptide ligand with great translational potential in tumor-specific imaging and chemotherapy drug delivery to the tumor sites while sparing the normal tissues, which is a thought-provoking technique to incorporate *A. platensis* IM femtoliposomes formulation with CRC-9 ligand to fight against colorectal cancer following the theranostic liposome formulation technique, but with some modification to obtain sustained release femtoliposomes incorporated with *A. platensis* in the body.

According to Zhai *et al.* [164], their work on chemotherapeutic docetaxel presented that transferrin (Tf)-targeted liposomes were an effective delivery system that has been utilized against breast, colon, ovarian, head, neck and non-small-cell lung cancer for their treatment. The expression of transferrin receptor (TfR) is higher in tumor cells as compared with normal cells and is associated with the increased iron demand in rapidly proliferating cancer cells, as well as, folate receptors were expressed. So with a folic acid-based liposomal system incorporated with folate ligands, could couple with folate receptors and aid the liposome to penetrate through the cell surface via the cell-penetrating peptide (CPP) [152,165,166]. This will be implemented in *A. platensis* stealth femtoliposome-CRC-9 specific IM formulation.

**Figure 5.**
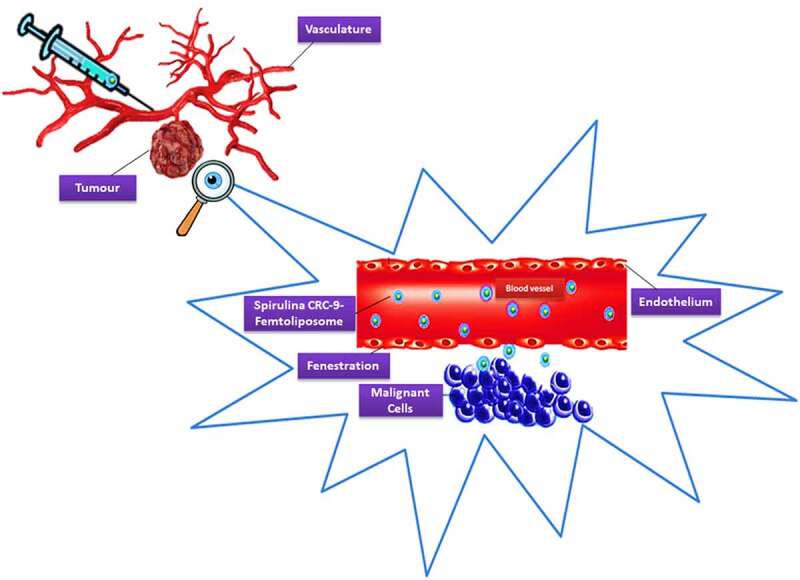
Spirulina stealth femtoliposomes-CRC-9 ligand-specific formulation flow in the blood vessel to reach tumors.

The *A. platensis* aqueous extract IM femtoliposomes formulation will be added to PEG solution to have a coated layer on top of it to obtain a PEGylated (stealth) femtoliposomes incorporated with *A. platensis* formulation then adds the CRC-9 ligand on the top of the stealth femtoliposomes. Immordino *et al.* [167] noted that PEG presence on the liposomal carrier surface increased blood-circulation time while minimizing stealth liposome absorption by the mononuclear phagocyte system. This technology’s effect is that many liposomes encapsulating active molecules have been formulated with high target efficiency and activity. Furthermore, according to Deshpande *et al.* [154], the added PEG improvises a steric stabilization effect once it is added to the surface of liposomes. PEG molecules modify the surface of the liposomes with a protective hydrophilic layer that prevents the blood components from aggregating and interacting with the liposomes, as illustrated in Figure 5.

**Figure 6.**
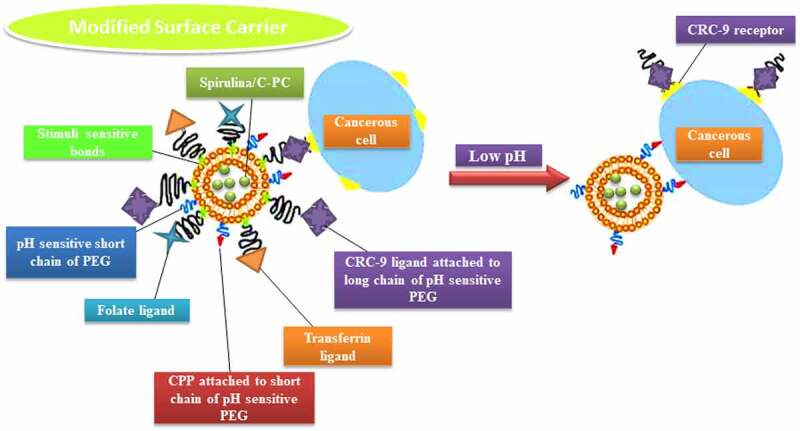
Formulation reaction under a low pH environment.

**Figure 7.**
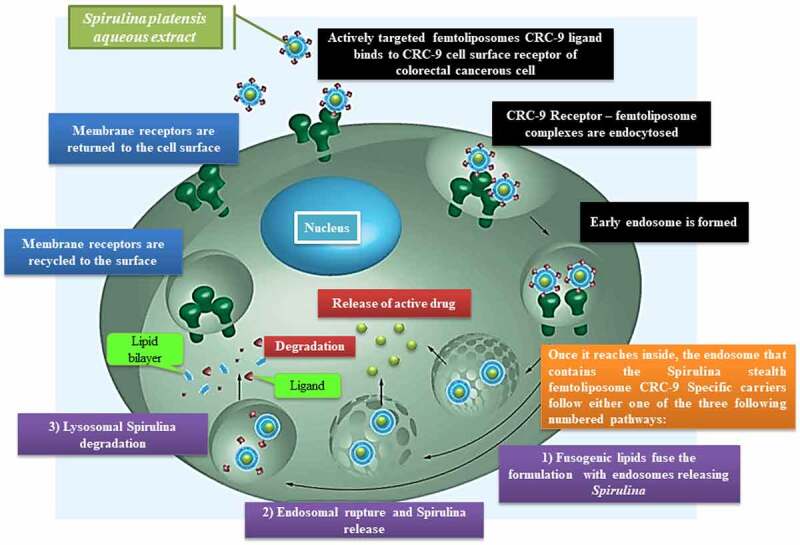
Spirulina stealth femtoliposomes-CRC-9 ligand-specific formulation mode of action.

**Figure 8.**
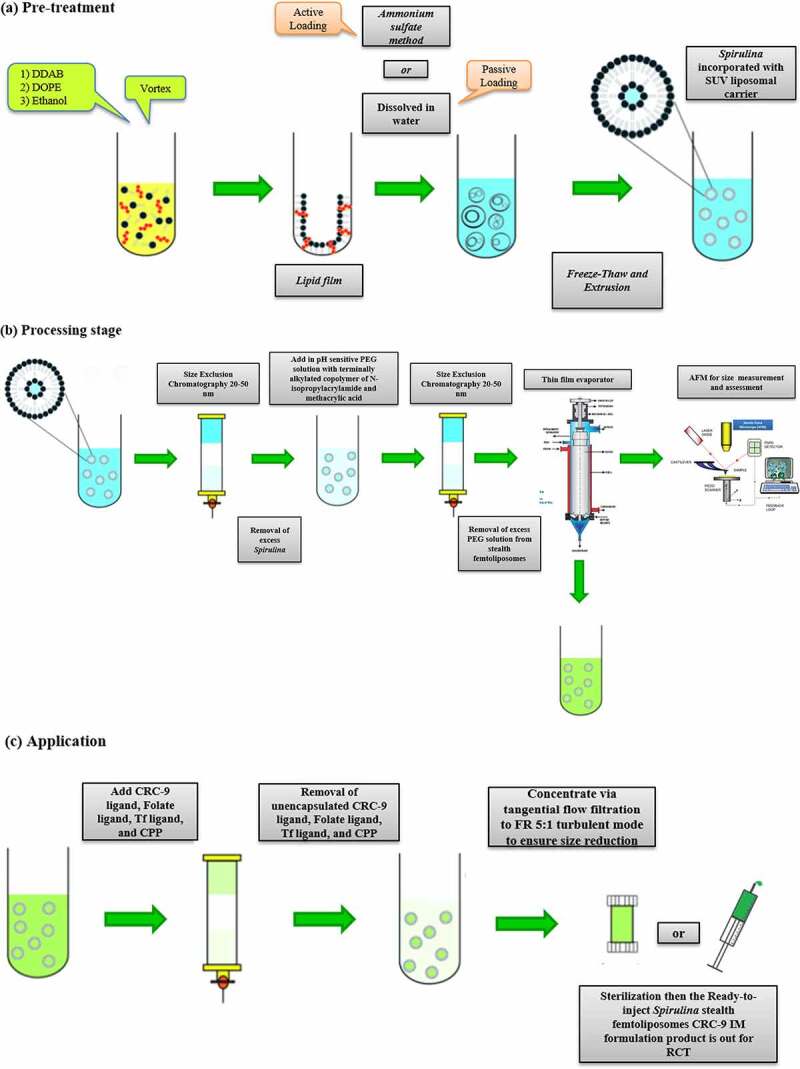
The procedures to produce a purified *A. platensis* stealth femtoliposomes-CRC-9 ligand-specific IM formulation can be separated into three stages (a) Pre-treatment (b) Processing stage (c) Application.

According to Arias [168], the pH-sensitive components fuse with the endovacuolar membrane after endocytosis. Subsequently, the carriers emit their contents in the cytoplasm after the reaction with low endosomal pH, as illustrated in Figures 6 and 7. Thus, this copolymer facilitates liposome destabilization and drug release in compartments with decreased pH values. The formulation could be stored in a vial or stored in a pre-filled syringe, labeled appropriately and preserved at 4°C [169]. The overall summary of the procedure is illustrated in Figure 8.

### 9.5. Commercialization

It is doable to make an IM parenteral dosage form of *A. platensis* and be manufactured and sold. However, some clinical studies and formulation adjustments should be considered and approved first. To conceive the first *A. platensis* IM injection with the highest quality of production following the current good manufacturing process (cGMP), a new drug delivery technique using stealth femtoliposomes and to achieve high therapeutic and pharmaceutical functions with minor side effects.

**Figure 9.**
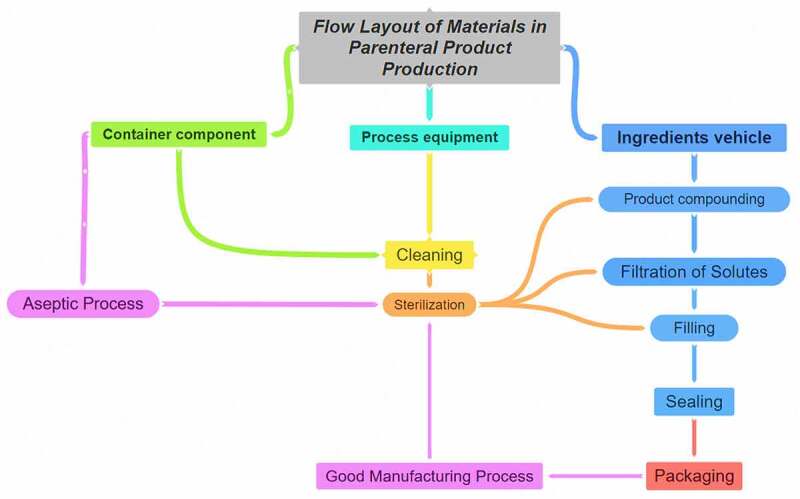
The schematic flow diagram of the industrial parenteral sequential process.

A good understanding of aseptic controls is essential for patient safety and may assist in guiding the process from candidate selection to process development. Cheminformatics [170], design of experiments, critical process parameters, critical material attributes, and critical quality attributes are considered the foundation for effective commercialization. Furthermore, for biologics scale-up production it requires a bioinformatics background on the gene of interest followed by genomics and proteomics studies to identify and select potential highly expressed genes for macroscale production, which interestingly could be achieved using microalgae as mentioned in the following references [171,172]. The bigger title for an efficient manufacturing scheme is known as scale-up with a delicate quality by design approach [173], all the beforementioned factors help to minimize the selection process to determine the scalability and robust development process for selected candidates. The risks of various approaches can be rapidly assessed with complementary analytical techniques, such as HPLC [174]. Suppose a product cannot be terminally sterilized, for instance, via steam or irradiation. In that case, the reliance on a wholly-aseptic compounding and filling process carries a significant risk of damaging the product [175]. Since the work core is on liposomal formulation, which is mainly used when the drug is poorly soluble or hydrophilic [158,176]. According to Savla *et al.* [176], this means that the product has to be assessed for suitability using high shear, high pressure, or ever-maturing microfluidics processes. The whole process layout is presented in Figure 9 [177] for manufacturing a parenteral product.

## 10. ‘Advanced Spirulina’ tablets

### 10.1. Why tablets?

The utilization of tablets is a sensible choice because of the ease of transportation and consumption flexibility. On top of this, the Objective Structured Clinical Examinations (OSCEs) consider tablets a safer mode of administration than other administrations due to the low rate of error that would be obtained. The OSCEs analyze the formulation level of functionality aided with satisfaction, the subjectively recognized amount of effort, the general impressions and evaluations of tablet-based administration [178].

### 10.2. Suggested enteric polymer for application

Generally, basic drugs will be absorbed in the intestines, while acidic drugs will be absorbed in the stomach [179]. The drugs or supplements will mostly be in neutral form when absorbed. Therefore, it depends on where the drug or supplement will disintegrate; different internal organ parts have different pH [180,181]. In the stomach, the pH is acidic from the 0-2 pH range, while in the intestines is alkaline from pH 6 and above. Some drugs or supplements are designed to be absorbed in the stomach and others in the intestines, depending on the nature of the drugs or supplements’ characteristics [181,182].

Spirulina possesses pH 6.93 ± 0.12 alkaline characteristics in nature [35] that requires a pH medium close to Spirulina’s pH, whereby the difference between both the organ environment pH that it will disintegrate at and Spirulina’s pH itself has to be close to each other; to have a less charged ions and become closer to neutral, so it would be accessible in offering a higher chance for the drug or supplement to penetrate through the phospholipid bilayer membrane of the organ wall [179,183]. Spirulina tablets nowadays disintegrate in the stomach, which scientifically is against Spirulina’s natural pH, and this would result in strong ionization (almost 99% ionization and 1% unionized) due to the significant difference of almost 2 pH units between the alkaline pka of Spirulina and the pH of the stomach. Hence, if the Spirulina’s constituents are not neutral or less charged, then this assures that the constituents in Spirulina formulation are not absorbed efficiently. Moreover, the body will not benefit from it because ionized compounds are less lipophilic and are less able to pass via a lipid bilayer [183,184].

Compared to if the constituents are disintegrated in the intestine, whereby the pH environment is alkaline, *A. platensis* becomes less charged and almost neutral to be almost absorbed to increase its bioavailability and chance for the body to benefit from *A. platensis’* functions [185]. This is a great chance to expose more results from *A. platensis* with a better therapeutic effect than it is already showing. It would be more reactive and efficient to expose their therapeutic and pharmaceutical functions if the amended ‘Advanced Spirulina’ tablets are utilized rather than those present in the market. A comparative study could be performed on them after being distributed publicly.

For instance, some *A. platensis* products, are written on the packaging container of Spirulina tablets products, particularly the ancillary supplement label instructions. It is required per dose for an adult to consume 6 tablets a day, whereby each tablet weighs 250 mg, including excipients. The active ingredient, which is *Arthrospira* (Spirulina) *platensis* in each tablet, weighs 240.75 mg [72]. The ‘Advanced Spirulina’ tablets will have better chemical and physical characteristics aided with fewer tablets ingested per dose and more kinetically sustained in the bloodstream.

The advantage of using a cellulose acetate phthalate (CAP) polymer chain; is that it has a free hydroxyl (-OH) group in each glucose unit. The basis of an enteric character in each glucose unit; is to have around 50% of its composition are acylated by electrophilic substitution and 25% are esterified by 1 or 2 carboxylic acid groups of the phthalate moiety via nucleophilic substitution 2 (SN_2_). This makes it suitable to use it as the first layer to delay the release of *A. platensis* from the tablet core by coating on the top of the ‘Advanced Spirulina’ tablets with DR polymer to encounter and resist the low pH in the stomach and become soluble in gastric fluids. This happens because the carboxylic acid (–COOH) group will only ionize in pH above 5.5, such as in aqueous environments whereby it induces one of the carboxylic acid groups that are free to react and the 2^nd^ carboxylic acid (–COOH) group for it to produce salt. One example of polymers that follow the same concept such as methacrylic acid-co-ethyl acrylate polymer derived from CAP that EUDRAGIT® Company manufactures. The polymer trade name is called L 30 D-55 [186].

**Figure 10.**
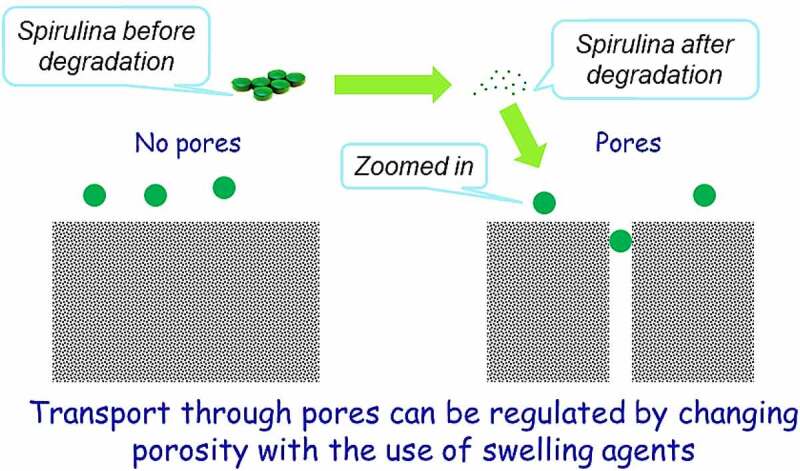
Ordered diffusion of Spirulina constituents via permeable pores in polymers.

Drugs diffuse through polymers by one of these two routes either via the polymer’s pores or directly diffuse through the polymer and achieve an SR property. The best choice to optimize Spirulina tablets is to induce a diffusion pathway through the pores permeant of the hydroxypropyl methyl-cellulose phthalate (HPMC) 2910 polymer resulting in an ordered release of the constituents, as illustrated in Figure 10.

The characteristics required to achieve a good, sustained release of Spirulina from the HPMC 2910 SR polymer are in the following:

1. Polymer chains have to move apart from each other to allow diffusion, which is more difficult in polymers than in simple liquids
2. Diffusion in polymers can only take place if there is enough free volume, for instance, spaces between the polymer molecules
3. True diffusion in polymers only occurs at temperatures above the glass transition temperature (T_g_) because below T_g_ this will be considered as an anomalous diffusion
4. Governed by Fick’s first law from high to low concentration gradient, down the concentration gradient
5. Polymer molecular weight
  - Increased molecular weight (MW) thus reduces diffusivity
  - Generally more difficult for polymer molecules to separate if molecular weight greater
6. Degree of crosslinking
  - Increased crosslinking thus reduces diffusivity
7. Plasticizers
  - The presence of plasticizers lowers T_g_, thus increasing diffusivity
8. Fillers/opacifiers/pigments
  - Reduced deformation, thus reduces diffusivity
  - Sometimes overusing could lead to an obstacle effect
9. Crystallinity
  - Crystalline regions present an impermeable barrier to most molecules
  - Increased crystallinity thus reduces diffusivity
10. The molecular size of permeant
  - Larger permeants require more free volume and consequently more polymer movement to diffuse; hence diffusion is reduced
11. Properties of permeant
  - Relative hydrophilicity

The most suitable area for *A. platensis* to disintegrate and the constituents are efficiently absorbed through the intestines wall and used as a targeting strategy for drug delivery in the intestines because it exhibits a relatively higher pH than the upper GI tract. Since the best decision taken is to target the ileum and to sustain the release of *A. platensis* till the rectum in the intestine, a DR polymer should be utilized to be the first layer on top of the ‘Advanced Spirulina’ tablets, such as methacrylic acid-co-ethyl acrylate derived from CAP to avoid ionization of constituents after the delay release polymer from EUDRAGIT® is separated from the ‘Advanced Spirulina’ tablets and nothing from *A. platensis’* constituents are harmed. Utilizing a designed alkaline pH-dependent polymer as the second layer to coat the tablets, such as HPMCP polymer for colon-targeted drug delivery system, will be the most appropriate opted option to release the *A. platensis’* constituents sustainably at zero-order kinetics from the tablet core the moment it starts to flow from the duodenum at pH 6 to the small intestine at pH ranges from 6.5 to 7.5, then finally to the ileum at pH 7.4 [181,187]. If the purified C-PC was to be delivered, it would be highly recommended to be deposited at the duodenum site since the isoelectric pH of C-PC is 5.8. The duodenum pH is 6, which means that it is favorably to have a similar pH to achieve a neutral charge of C-PC protein and to maximize its bioavailability and therapeutic effect [40,66,126].

The EUDRAGIT® polymer dissolves around the cecum area because of the sudden pH drops to around pH 5.7 [181]. The SR polymers will be applied and used as the second polymer underneath the DR polymer, for instance, HPMCP 2910 from EUDRAGIT® Company for engineered drug delivery systems. According to Tahara *et al.* [188], SR polymer incorporated with tablet matrices prepared with HPMC 2910 polymers was investigated to define the conditions for selecting suitable polymers for SR formulation development. The tablet erosion rate can also be adjusted by choosing HPMC polymer viscosity or mixing HPMC with different viscosity and variation percentages of monomers. Since *A. platensis* is water-soluble, opting for high viscous HPMC polymer will be the best decision with small pores to sustain *A. platensis* constituents’ release throughout the duodenum till the rectum. Table 5 shows the ionisiation of substances affected by the pH of different areas in the human body.

**Table 5.** The ‘Advanced Spirulina’ tablets pathway order through the GI tract with their pH and ionization analysis.

Pathway order	Organ and Sub-organ parts	pH	pH difference with Spirulina pH 6.93 ± 0.12 in nature	Ionization %	
				Ionized	Unionized
1	Esophagus	1.0 – 4.0	DR polymer present	0	100
2	Stomach	0 – 2.0	DR polymer present	0	100
3	Duodenum	6.0	0.93 ± 0.12	90	10
4	Proximal small intestine	6.5	0.43 ± 0.12	50	50
5	Distal small intestine	7.5	0.57 ± 0.12	50	50
6	Ileum	7.4	0.47 ± 0.12	50	50
7	Cecum	5.7	1.23 ± 0.12	90	10
8	Ascending colon	5.7	1.23 ± 0.12	90	10
9	Transverse colon	6.6	0.33 ± 0.12	50	50
10	Descending colon	7.0	0.07 ± 0.12	50	50
11	Rectum	6.7	0.23 ± 0.12	50	50

**Figure 11.**
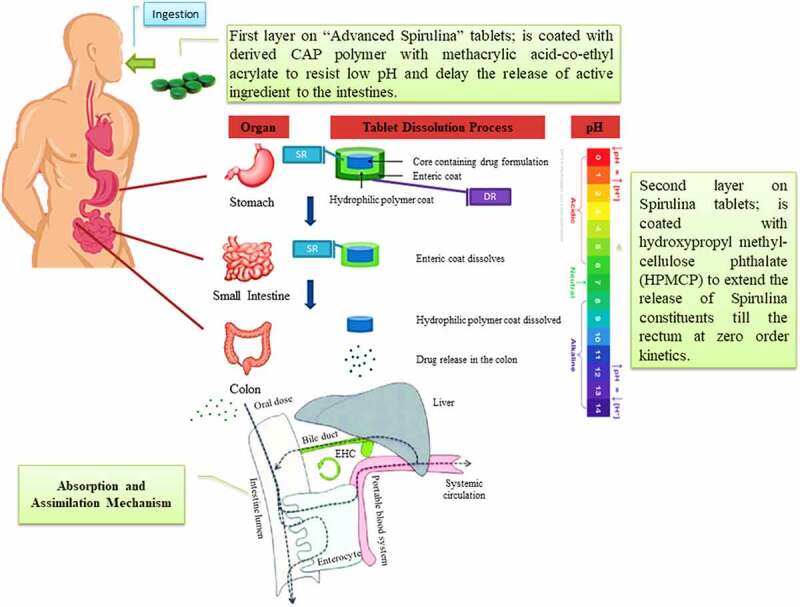
‘Advanced Spirulina’ tablet dissolution mechanism for the release of *A. platensis’* constituents in the intestine.

**Figure 12.**
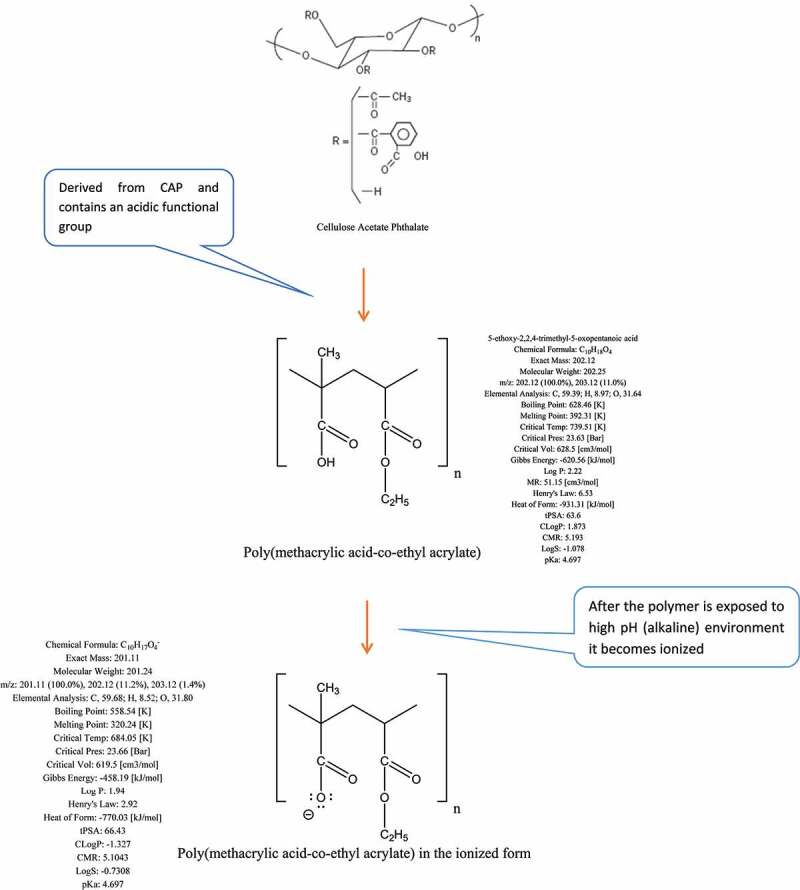
The analysis of the methacrylic acid co-ethyl acrylate polymer’s chemical properties before and after ionization.

**Figure 13.**
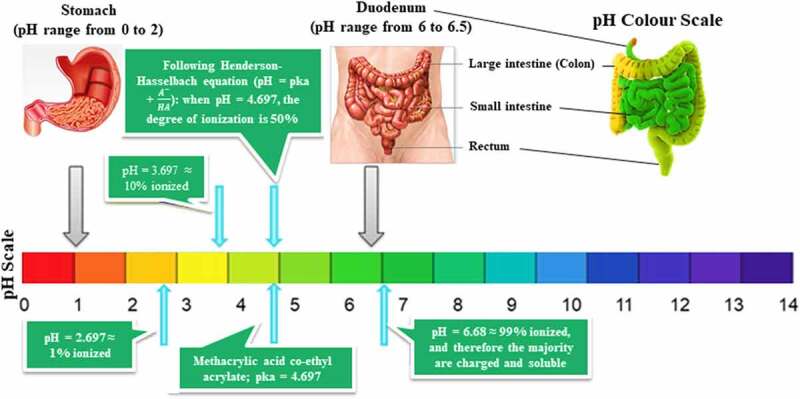
Methacrylic acid co-ethyl acrylate polymer ionization analysis at different pH using the Henderson-Hasselbach equation.

The polymer’s general characteristics are in the illustrative figures: To be capable of withstanding the stomach’s low pH and enabling the release of *A. platensis’* constituents at the start of the duodenum to the rectum. To achieve this, ‘Advanced Spirulina’ tablets will be coated primarily by a DR polymer that will be degraded in the stomach without touching the secondary polymer just to pass from the stomach area to the intestine, as illustrated systematically in Figure 11. Once it safely passes from the stomach, the partially neutralized DR L-30-D-55 polymer on the top will ionize almost 99% at alkaline pH using the Henderson-Hasselbach equation and will not be absorbed, as illustrated in Figure 12 (Using Chemdraw professional office software) and Figure 13. Then, a secondary HPMC 2910 SR enteric polymer is located on the top of the tablet core; to release *A. platensis’* constituents to the rectum.

According to Zu *et al.* [189], their research team investigated the drug release percentage from different L-30-D-55 (poly (MA-EA)) polymers in acidic and buffer phases. The drug release percentages were recorded, particularly L-30-D-55 type of polymers from partially neutralized and unneutralized poly (MA-EA) coated pellet-filled capsules. The author suggested that in the initial step of stability studies, as illustrated in Figure 12 [189], the physical difference between neutralized and unneutralized poly (MA-EA) coating films had no significant effect on the drug release. Both polymers exhibited similar dissolution profiles in the buffer media, and both coating systems effectively hindered the drug release in acidic media. However, substantial delay releases of the drug from the pellet-filled capsules coated with unneutralized poly (MA-EA) in the buffer stage at pH 6.8 was observed after accelerated storage at 60°C. Since it is a pH-dependent polymer, it will still possess the same enteric polymer characteristics to dissociate in the intestines, not only for *A. platensis* but this modified polymer could also be used and applied to any drug or supplement that would be absorbed better in the intestine rather than in the stomach.

Eventually, it gradually disintegrates through the journey from the small intestine to the colon by coating the ‘Advanced Spirulina’ tablets with a secondary sustained release polymer that releases Spirulina constituents at zero kinetic order. These polymers could be personalized and processed by EUDRAGIT® Company uses polymers for drug delivery purposes that provide pH-dependent drug release and mucoadhesive synthetic copolymers for colonic drug delivery. As a result, the dissolution of the polymer coating *A. platensis* will exist when it reaches the pH threshold range of 6.0–7.0 and is expected to delay the dissolution action and prevent the premature release of *A. platensis’* constituents in the upper GI tract before reaching colonic sites using partially neutralized ƒ-DR L-30 D-55. After that, using a secondary HPMC 2910 SR enteric polymer would sustainably release *A. platensis’* constituents throughout the lower GI tract journey at zero kinetic order. Concurrently, perform a pharmacokinetic study is required to investigate the time required for ‘Advanced Spirulina’ tablets to get metabolized and excreted from the body using these polymers.

Some limitations have been encountered; for instance, drug release significant variability, and various inter-and intra-subject alterations in critical parameters that eventually could lead to the failure of *in vivo* tests. These parameters include the volume of fluids, motility, pH, and GI transit times. On top of this, a significant variation in pH ranges might be experienced because of the diet, water intake, disease state and microbial metabolism. Perhaps, with proper regulation of these parameters using these new modified polymers, then this should avoid any potential failures during experimentation [190,191].

By using the Henderson-Hasselbach equation and applying the equation using the data acquired from the hospitalized patients with colorectal cancer that was investigated by Newmark and Lupton [192], have found that the mean fecal pH is 8.02, which was significantly higher than 6.6 for control subjects [192] that will result for the ‘Advanced Spirulina’ tablets to almost 90% ionize at the colorectal region since the assumed pH is as the fresh stool pH recorded. Therefore, this could target the cancerous cell in the colorectal region because *A. platensis* will be ionized and will not be absorbed by the gut wall to the bloodstream. Similarly, the mean stool pH of a Seventh-Day Adventist population was 6.5 compared to 6.7 for controls and 7.0 for subjects with colon cancer [192], whilst if the pH is 7, then it will be almost the same and similar to the pka of *A. platensis*; as a result, 50% ionization of *A. platensis’* constituents will exist.

## 11. Available formulations in the market

Several *A. platensis*-based products are available in the US as food (mostly utilized as food coloring) or dietary supplements and in different dosage forms, including powder, capsules and tablets [51]. There is also a potential to create a cream dosage form and one of its ingredients is *A. platensis* based upon these sources [193,194]. The molecules in *A. platensis* are very delicate to alterations in light, extraction/purification solvents, pH, temperature and the different quantities of total phycocyanins (C-PC, APC, and phycoerythrin (PE)) could explain and describe different biological efficacies [195].

According to Böcker *et al.* [196], their experimental research data results portrayed almost 80% of phycocyanin degradation at pH 5 and pH 7 in an aqueous solution with an exposure of 3 × 10^5^ lux for 24 hours of light. Phycocyanin protein is unstable to light and heat in an aqueous solution and insoluble in an acidic solution at pH 3. This protein denatures at pH 5 and 7 with temperatures > 45°C, resulting in the change of color. According to Jespersen *et al.* [197], even though C-PC’s possibility is high for implementations in medicine, biotechnology and the food industry, one of the main limiting factors for its successful application is stability, which could be surmountable with the application of suitable strategic storage techniques.

For tablet dosage forms located and distributed in high humidity countries such as Malaysia. Countries with high humidity will require a personalized variation of polymers to resist hygroscopic effect because of the high moisture content in the atmosphere it is stored, to be dispensed to patients safely with dry and stable tablet conditions [198]. So, it is recommended to add desiccant silica gel in the container to avoid the potential degradation of tablets by absorbing moisture content in the container’s atmosphere to keep tablets intact [199]. According to each country’s weather, a specialized good distribution practice (GDP) guideline is required for storage scientists to follow to achieve a high safety storage level for drugs or supplements [200].

## 12. Conclusion

Although there is still a need for further improvements, overcoming pathophysiological variability will remain more helpful than the pH-dependent system solely in different release-triggering mechanisms using integrated systems. Besides nano-/micro-particles, this innovation can potentially be used in delivering drugs or supplements using Femto-particles that provide better distribution, flowability, bioavailability, therapeutic and pharmaceutical effects, and less interaction with the body’s microsystems interventions. The stealth femtoliposomes can be used in IM dosage form and film coating femtopolymers used for the tablet’s dosage form. The delivery system also holds an excellent possibility for specifically labeling and localizing inflamed intestinal tissues, improving drug uptake, and could be applied to several drug or supplement techniques. Eventually, many formulations that have integrated a pH-dependent system with particle size reduction have been developed for intestinal-targeted drug or supplement delivery. More clinical trials should be done on *A. platensis* as extracts, constituents and ingredients due to the aforementioned comprehensive research findings proving its good medicinal characteristics and properties against diseases. Positive health-related reports are not integrated deeply and accurately enough, putting limitations on its application as a drug. The healthcare system requires new innovative delivery techniques of drugs or supplements that have been scientifically proven to reveal their hidden maximal therapeutic outcomes. Indeed, there is no advantage to discovering new drugs or supplements without delivering them appropriately.
